# Identification of novel MiRNAs and MiRNA expression profiling during grain development in indica rice

**DOI:** 10.1186/1471-2164-13-264

**Published:** 2012-06-21

**Authors:** Ying Lan, Ning Su, Yi Shen, Rongzhi Zhang, Fuqing Wu, Zhijun Cheng, Jiulin Wang, Xin Zhang, Xiupin Guo, Cailin Lei, Jie Wang, Ling Jiang, Long Mao, Jianmin Wan

**Affiliations:** 1 National Key Laboratory of Crop Genetics and Germplasm Enhancement/Jiangsu Plant Gene Engineering Research Center, Nanjing Agricultural University, Nanjing, 210095, China; 2 National Key Facility for Crop Gene Resources and Genetic Improvement, Institute of Crop Sciences, Chinese Academy of Agriculture Sciences, Beijing, 100081, China

**Keywords:** miRNA, Grain filling, Indica rice

## Abstract

**Background:**

MicroRNAs (miRNAs) modulate gene expression in different tissues and at diverse developmental stages, including grain development in japonica rice. To identify novel miRNAs in indica rice and to study their expression patterns during the entire grain filling process, small RNAs from all stages of grain development were sequenced and their expression patterns were studied using customized miRNA chips.

**Results:**

A total of 21 conserved and 91 non-conserved miRNA families were found in developing indica grains. We also discovered 11 potential novel miRNAs based on the presence of their miRNA*s. Expression patterns of these identified miRNAs were analyzed using customized miRNA chips. The results showed that during the filling phase about half of the detected miRNAs were up-regulated, whereas the remainder were down-regulated. Predicted targets of differentially expressed miRNAs may participate in carbohydrate metabolism, hormone signaling and pathways associated with seed maturity, suggesting potentially important roles in rice grain development.

**Conclusions:**

This study is the first genome-wide investigation of miRNAs during the grain-filling phase of an indica variety of rice. The novel miRNAs identified might be involved in new miRNA regulatory pathways for grain development. The complexity of these miRNAs and their targets and interactions require further study to obtain a better understanding of the molecular mechanisms underlying grain development.

## Background

Small RNAs, microRNAs (miRNAs) and short interfering RNAs (siRNAs) are important gene-regulatory molecules at both the transcriptional and post-transcriptional levels in eukaryotic cells [1]. Plant miRNAs are derived from single RNA molecules. Primary RNA precursors (pri-miRNA) can form imperfect stem-loop structures where a miRNA/miRNA* duplex is processed from the stem by Dicer-like 1 (DCL1) or DCL4 [2-4]. Plant miRNAs negatively regulate their cognate mRNAs by fully or partly binding to complementary sites. After being methylated at the 3′ end by Hua Enhancer 1 (HEN1) [5], the mature miRNA with a length of 20–24 nucleotides (nt) is loaded onto the RNA-induced silencing complex (RISC) to direct the cleavage of its mRNA targets based on extensive complementarity. Plant miRNAs predominantly modulate their targets by mRNA cleavage, and some classes of 24 nt miRNAs direct cytosine DNA methylation at target genes to regulate their expression [6-8]. More recently, miRNA regulation of gene expression *via* DNA methylation and chromatin modification has been suggested [9,10]. The nearly perfect complementarity between miRNAs and their target sites makes it possible to predict their targets by computational approaches. miRNAs were shown to regulate genes involved in basic developmental processes, such as leaf development, flowering time, organ polarity and auxin signaling [11,12], as well as stress responses and disease resistance [13,14].

High-throughput sequencing technologies allow the discovery of a large set of diverse plant miRNAs. Thousands of miRNAs have been identified in different plant species, rapidly enlarging the identified plant miRNA pool, including miRNAs from different tissues or developmental stages. Based on the recent version of miRBase (http://www.mirbase.org/), over 400 miRNAs have been identified in rice. Among them, 21 miRNA families are evolutionarily conserved between Arabidopsis and rice [15-18]. Some of the miRNAs are conserved only among closely related monocots, suggesting the emergence of novel miRNAs after divergence of monocots and dicots [19,20].

As one of the most important food sources for the world’s population, rice is also an ideal model plant representing cereal crops. The grain-filling phase is a major stage of plant development that largely determines yield and quality [21]. During this process, all resources of the plant contribute toward a steady rate of starch accumulation in the storage units of rice grains [22,23]. In general, the grain development process can be divided into early development and filling phases. The former is characterized by high biosynthetic activity in grain formation when the total dry matter starts to increase and endosperm starch begins to accumulate rapidly in the seed (6–17 days after flowering, or DAF), whereas during the latter phase (from 18 DAF) the grain usually exhibits a slower increase in dry weight until maximum values are reached and grain weight becomes constant. Global gene expression profiling studies of mRNAs have shown that many genes in multiple pathways participate in grain filling processes, such as those involved in nutrient synthesis, starch synthesis and transport [24,25].

On the other hand, miRNAs were identified as preferentially expressed in various rice organs, including leaf, root, panicle and stem, as well as in seedlings under various stress treatments [17,26,27]. A number of studies were also carried out on small RNAs in the grains of japonica varieties [26,27]. Some miRNAs were preferentially expressed in early developing rice grains, such as 1–10 DAF and 3–12 DAF [28,29], suggesting regulatory roles of miRNAs during grain development. These studies, mainly in subspecies of japonica, also identified significant numbers of both conserved and non-conserved miRNAs. We report here the generation and sequencing of a small RNA library from grain tissues sampled during the entire grain filling stage of an indica cultivar. In addition to numerous conserved miRNAs, we identified 11 novel miRNAs. Subsequently, a customized miRNA chip was generated and miRNA expression profiling was studied using RNA samples from grains of each of the three filling stages: viz. milk-ripe (6–12 DAF), soft-dough (13–17 DAF), and hard-dough (18–20 DAF). Our results showed that most of the widely conserved miRNAs were down-regulated during grain develop-ment whereas rice or grass-specific miRNAs were up-regulated. The targets of differentially expressed miRNAs appeared to be involved in multiple biological processes, such as carbohydrate metabolism, hormone signaling and pathways associated with seed maturity, suggesting that rice miRNAs may play important roles during grain development.

## Results

### Small RNA populations at the grain filling stage

**Figure 1:**
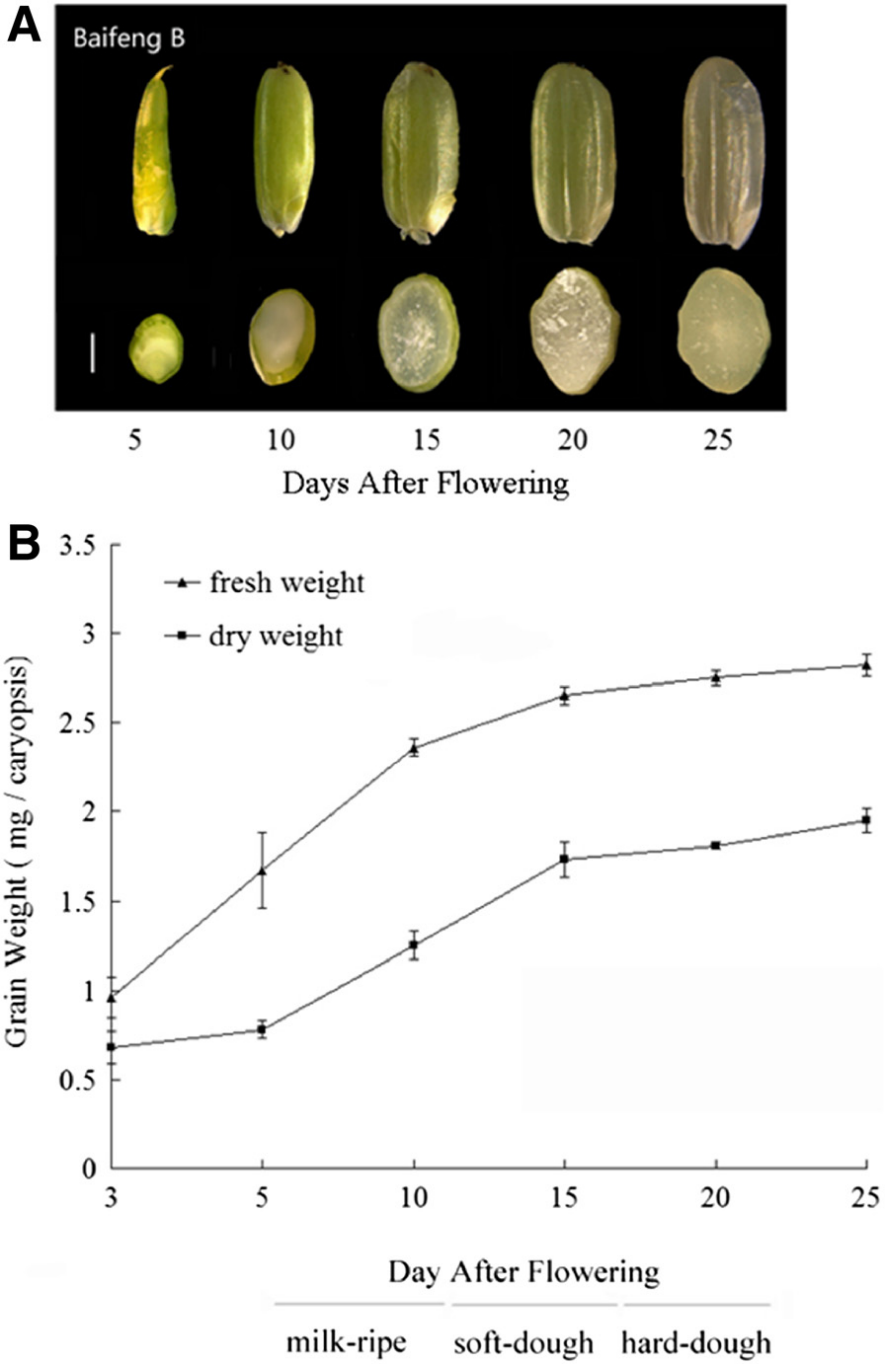
**Grain development and filling of indica rice caryopses.** Developmental changes in caryopses. Scale bars represent 1 mm. (**B**) Grain weight per kernel of fresh and dry caryopses during grain-filling. The experiment was repeated four times and bars represent the standard error.

We measured the fresh and dry grain weights of rice cultivar, Baifeng B, an indica landrace, at several stages of grain-filling (3, 5, 10, 15, 20, and 25 DAF). The fresh weights began to increase from 3 DAF; dry matter accumulation became faster from 5 DAF and reached highest levels at about 25 DAF (Figure 1). Morphological observations of developing rice seeds showed that the filling phase can be divided into three continuous filling stages. For Illumina sequencing, we isolated small RNAs from immature rice grains sampled at 5 DAF to 25 DAF. After removing low-quality reads, a total of 1,832,288 clean reads were obtained with 974,934 unique sequences. About 637,362 distinct reads (65.4%) were aligned to the ‘9311’ genome using short oligonucleotide alignment program (SOAP) [30] (Additional file 1). Among them, 21 nt and 24 nt small RNAs form the two largest groups, accounting for 22.3% and 50.5% of raw reads, respectively (Additional file 2). By comparison with miRNAs from miRBase v16.0, 102 known miRNAs (71.8%) were found in our dataset. All of these miRNAs, except for miR827, were members of 21 families that are conserved in diverse plant species (Additional file 3A). The abundance of miRNAs varied greatly. MiRNA families highly conserved across plant species, such as miR166, miR167, and miR168, were sequenced more than 10,000 times, whereas previously known stress-induced members, such as miR395 and miR399, were detected less than 10 times (Additional file 3A), indicating that tissue-specific expression patterns of miRNAs are related to their functions. In contrast, most rice- or monocot-specific miRNAs were detected with low read numbers, except for miR444 and miR528, which were represented by 3,917 and 6,305 copies, respectively.

There were significant variations in expression levels for members of the same family. For example, the abundance of the miR159 family varied from 9 (miR159c) to 7,113 (miR159a.2) reads. Similarly, the abundance of members of the miR166 (from 1 to 14,397 reads) and miR164 (from 12 to 6,871 reads) families were also highly variable (Additional file 3A). Twenty previously reported non-conserved miRNA families were not detected in our dataset. A major reason for this might be the limited low sequencing depth, at which the expression level of this group of miRNAs might have been too low to be detected in our library. Another factor may have been the different subspecies and cultivar used compared with previous work. We found that the locations of many miRNA reads varied within a ±2 nt range from the 5′ or 3′ ends of annotated miRNA sequences. Some of these variants even had similar reads compared with those annotated in miRBase. For example, the annotated miR1870 had 11 reads in our libraries, whereas the other 22 nt variants had 14 reads. Interestingly, some miRNA*s had higher read numbers than the corresponding miRNAs. For example, miR529* and miR2124* had more reads than their respective miRNAs, 135 vs 0 and 117 vs 1, respectively, suggesting that miRNA* may play a major role in these cases (Additional file 3A).

### Identification of 11 novel miRNAs in developing caryopses

**Figure 2:**
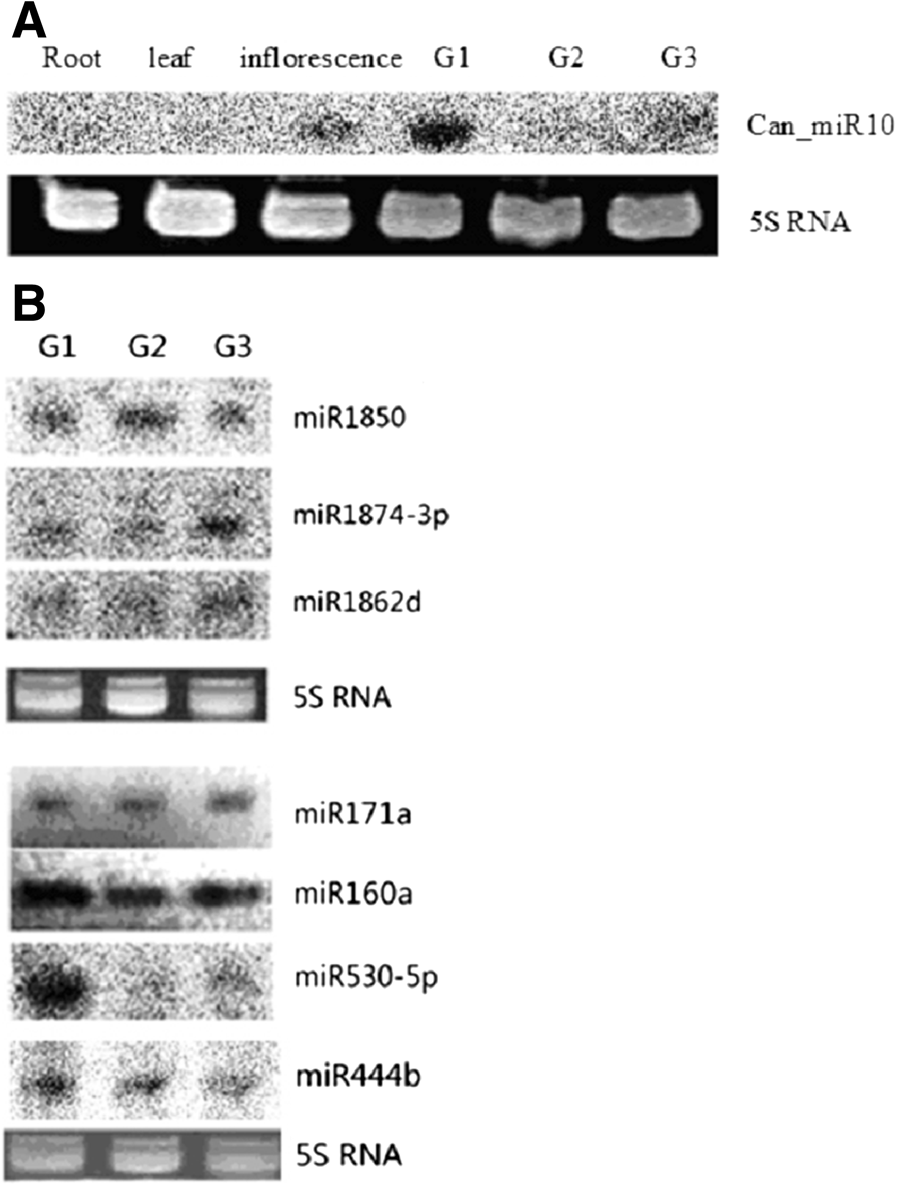
**Northern blot analysis of novel and registered rice miRNAs.** (**a**) Northern blots of novel miRNAs isolated from rice root, leaf, inflorescence and grains from three filling stages G1 (6-12DAF), G2 (13-17DAF) and G3 (18-20DAF). The blots were probed with ^32^P end-labeled oligonucleotides; (**b**) Blots of registered rice miRNAs isolated from unhusked rice grains from filling stages G1-G3. 5 S rRNA was used as a loading control.

To find novel miRNAs, we first mapped all the small RNAs to the sequenced indica cultivar “9311” genome because Baifeng B is an indica landrace. Secondary structures of sequences around the small RNAs were produced using Mfold. These putative miRNA precursors were then used to find miRNA*s, which are considered strong evidence for DCL1-derived products [31]. We found 11 regions that satisfied these criteria and considered them to be novel miRNA gene candidates (Table 1 and Additional file 4). Most novel miRNAs showed weak expression levels (sequencing frequency < 50). The reads for their miRNA*s were even lower. All of these newly identified miRNAs appeared to be rice-specific and had not been reported in other species. Most novel miRNAs were not detectable by northern blotting, except Can_miR 10 (Figure 2A), but all were confirmed by using more sensitive array analysis (see next section). Surprisingly, novel miRNAs discovered in previous deep sequencing of rice grain small RNAs were rarely present in our dataset. Among 39 novel miRNAs in the 1–10 DAF rice grain library [28], and another 26 reported by Xue et al. [29] in a 3–12 DAF rice grain library, only nine were detected in our library. These included miR1862d and miR1862e with relatively abundant expression levels of 181 and 122 reads, respectively, whereas the others (miR1849, miR1850, miR1856, miR1860, miR1861e, miR2098-3p and miR827b) were detected with expression levels of only one to five reads (Additional file 3B and 3 C). The lack of shared novel miRNAs could be: 1) due to our using indica cultivar Baifeng B whereas all previous studies were with subspecies japonica; 2) because the majority of the rice-specific miRNAs are expressed at very low levels, they might not have been detected at our sequencing depth.

**Table 1:** Predicted novel miRNAs

**Name**	**Sequence**	**Length**	**Abundance**	**Chromosome**	**Location**
Can_miR_01	ACGGAAAAUCAUGGCUGCACUUAA	24	26	1	Intergenic
Can_miR_02	AAUCAAGUUAGGAACCAUGCAAGU	24	2	6	3′UTR
Can_miR_03	ACUCUAUAUGAACUAAGAUCG	21	6	8	Intergenic
Can_miR_04	UUGGCUGCAUCCCGUUCUCCUC	22	7	4	Intergenic
Can_miR_05	AGCUGCCGACUCAUUCACCCA	21	30	1	Intergenic
Can_miR_06	UCUCUCUCUCCCUUGAAGGCU	21	3	11	Intergenic
Can_miR_07	AUGAAUGUAGGUAAUGCUAGAAAG	24	3	1	Intergenic
Can_miR_08	GAAUGAUCAAAGUUGGACACGAA	23	8	1	Intron
Can_miR_09	ACCUCAACAUGGUAUCAGAACUGG	24	8	7	Intron
Can_miR_10	UCAAUAGCGAUCAAGGCGGAC	21	3	7	Intergenic
Can_miR_11	UGGGAAAGGACCAUAAUACCCCUA	24	7	3	Intron

Targets of novel miRNAs were predicted and some appeared to be involved in the grain filling process (Additional file 5). For example, Can_miR_07 was predicted to target starch synthase II, which is preferentially expressed in the endosperm at the middle to later stages of grain filling and plays an important role in elon-gation of α-1,4 amylase chains [32,33]. Can_miR_04 and Can_miR_08 may target a ubiquitin-protein ligase gene and a carboxylate oxidase gene, which are known to be involved in cell death and fruit ripening progress [34], respectively. One target of Can_miR_06 is the growth-regulating factor gene (Os04g51190), which is also targeted by miR396 [35], indicating that multiple miRNAs may regulate the same gene family.

### MiRNA profile changes during grain filling

**Figure 3:**
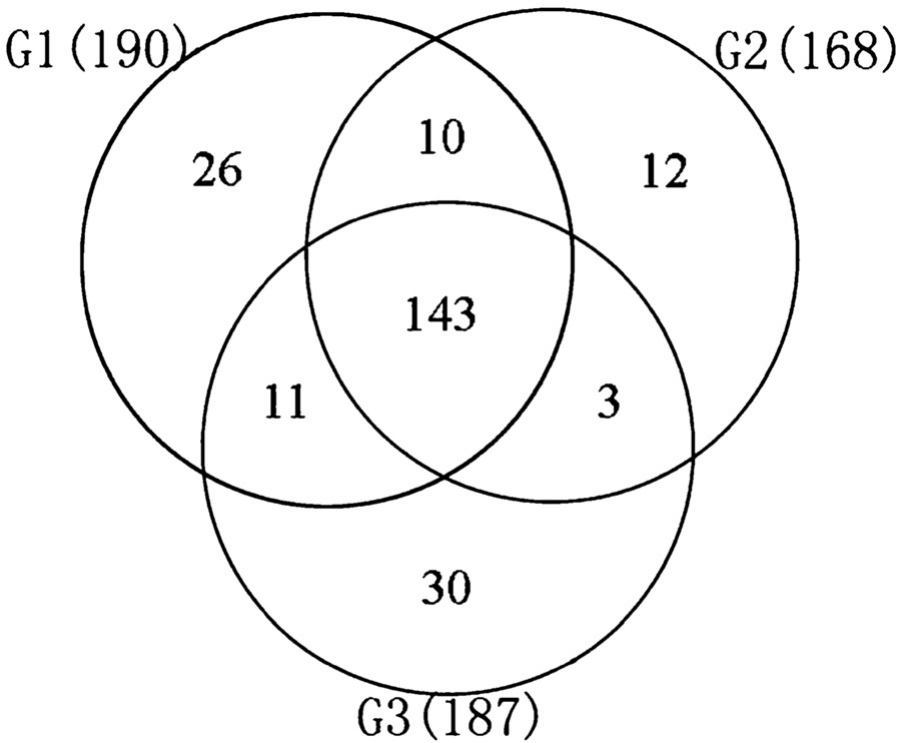
**Venn diagram of the differentially expressed miRNAs at the three stages of rice grain filling.** Numbers in parentheses are numbers of differentially expressed miRNAs at each stage.

To study the expression patterns of miRNAs during grain development, we generated miRNA chips containing 546 probes, and comprising 254 known miRNAs from miRBase version 13.0, the 11 newly identified candidates, and 50 controls. Small RNAs isolated from grains at the milk-ripe stage (6–12 DAF, or phase G1), the soft-dough stage (13–17 DAF, or phase G2), and the hard-dough stage (18–20 DAF, or phase G3) were hybridized to the miRNA chips. The raw signal values are provided in Additional file 6. As shown in Figure 3, 190, 168, and 187 miRNAs were detected above background levels (signal intensity >32) in G1, G2, and G3, respectively. Among them, 143 miRNAs were expressed in all three filling stages, whereas 26, 12, and 30 were specifically expressed in G1, G2, and G3, respectively. Most of the phase-specific miRNAs were newly identified, such as Can miR_11, which is expressed at G1 and G2, Can_miR02 and Can_miR03, which are expressed at G2 and G3, and Can_miR04 and Can_miR11*, which are detected only at G3 (Additional file 6C).

**Figure 4:**
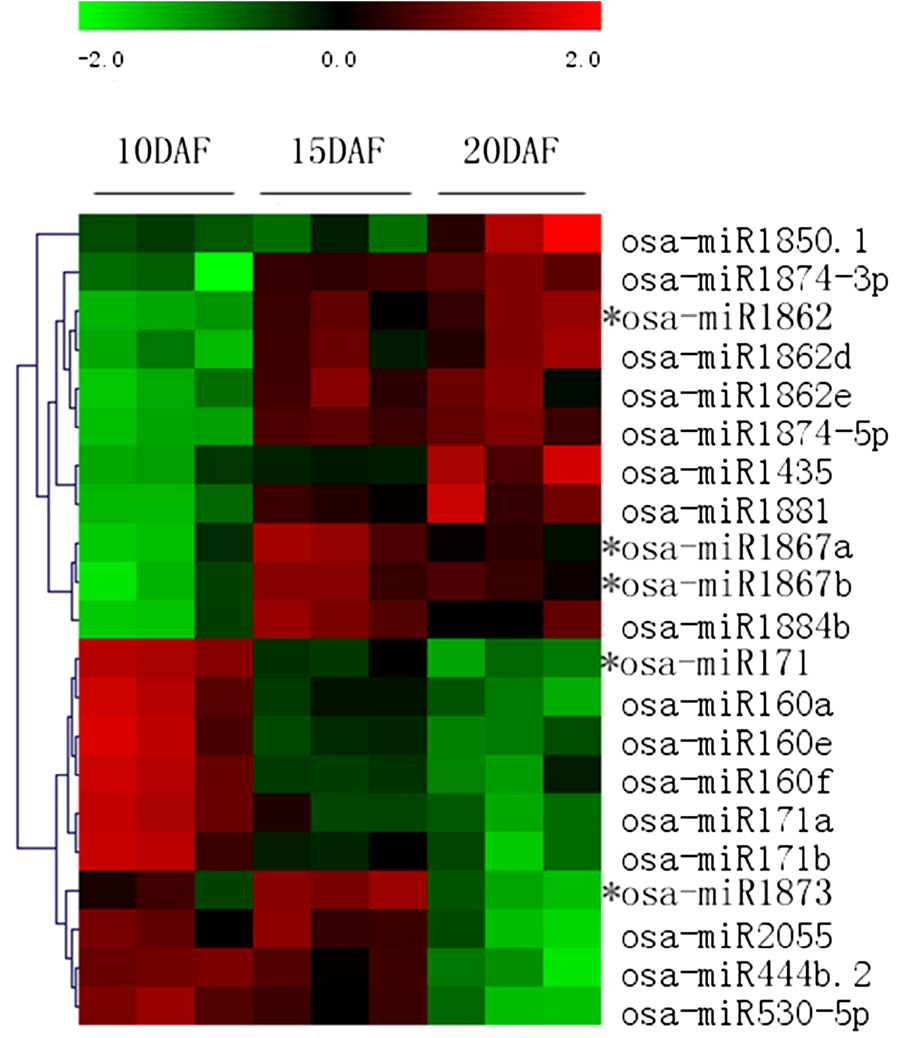
**Clustering of miRNAs differentially expressed during grain filling.** Comparison of miRNA levels obtained by chip hybridization (left panel) with signature abundances of miRNAs (right panel) obtained from data series in miRBase. Sequences with low abundance were filtered out. The bar represents the scale of relative miRNA expression (Log2). * indicates an miRNA variant from the same miRNA precursor that differs from the submitted sequence.

Using a relative intensity change of 2-fold or above between consecutive filling stages, the expression patterns of miRNAs were clustered (Table 2). As shown in Figure 4, 13 miRNA families included 18 members that were differentially expressed across the three filling stages. Nine members of seven miRNA families were up-regulated. The expression of miR1862 and miR1874 increased from G1 to G2, but remained largely unchanged from G2 to G3, whereas miR159, miR164 and miR1850 underwent rapid increases from G2 to G3. In contrast, nine members of six miRNA families were down-regulated. Among them, the expression of miR160, miR166, and miR171 declined rapidly from G1 to G2, whereas miR167, miR396, miR444 and miR530 gradually declined with advancing grain filling. The expression of miR2055 also declined rapidly from G2 to G3. Some of these expression patterns were consistent with results from northern blot assays. It seems that conserved miRNAs were mostly down-regulated whereas rice- or grass-specific miRNAs were up-regulated during the course of grain filling. As shown in Figure 2B, miR1862, miR1874 and miR1850 were significantly up-regulated, whereas miR171, miR160, miR444 and miR530 were down-regulated. The expression of miR2055 could not be confirmed probably because its expression level was too low.

**Table 2:** Expression data of miRNAs differentially expressed during the three filling stages

**Pattern of regulation**	**miRNA name**	**MiRNA expression signals (average)**^**b**^			**Fold change**^**b**^	**Fold change**^**b**^	**p-value**^**a,c**^
		**G1**	**G2**	**G3**	**(G2/G1)**	**(G3/G2)**	
Up	miR1850	56	53	293	0.95	5.53	7.40E-03
	miR1874-5p	349	1140	1266	3.27	1.11	2.29E-05
	miR1862e	133	378	384	2.84	1.02	3.69E-03
	miR1862d	247	526	656	2.13	1.25	3.17E-03
	miR1874-3p	2473	4684	5507	1.89	1.18	3.57E-03
	miR1435	86	138	357	1.60	2.59	2.24E-03
	miR1881	36	65	88	1.81	1.35	1.78E-03
	*miR1867	109	219	186	2.01	0.85	3.14E-03
	miR1884b	99	213	168	2.15	0.79	3.41E-03
Down	miR171a	118	58	42	0.49	0.72	1.13E-03
	miR171b-f	451	253	182	0.56	0.72	4.64E-03
	miR530-5p	4522	3286	1514	0.73	0.46	4.18E-04
	miR160f	125	38	31	0.30	0.82	1.27E-03
	miR160a-d	251	69	38	0.27	0.55	8.01E-04
	miR160e	275	68	48	0.25	0.71	1.16E-03
	miR444b.2	770	443	63	0.58	0.14	3.68E-04
	*miR1873	123	65	47	0.52	0.72	5.21E-05
	miR2055	138	140	97	1.01	0.69	5.23E-03

*: The probe sequence is on the same precursor of this miRNA, but had a ±2 nt range from the annotated miRNA sequence.

a: p-value < 0.01.

b: Mean levels averaged from three biological replicates; fold changes in miRNA levels were compared from the milky (G1) to hard dough (G3) stages and selected as less than 0.5 or more than 2.0 (from G1 to G3).

c: Signal values <100 (lower than three times the background signal) were not considered for variance analysis.

### MiRNA-mediated target mRNA cleavage and target expression patterns during grain filling

To further study the potential effects of differentially expressed miRNAs during grain filling, we computationally predicted their targets using the miRU program. Rapid amplification of 5′ cDNA ends (5′ RACE) was used to validate the cleavage events. As shown in Additional file 7A, most targets of conserved rice miRNAs, such as targets of miR160, miR166, miR171, miR444 and miR530, were annotated to be similar to those from other studies. However, the miR1435 target Os04g44354, a UDP-glucuronosyl transferase protein, was not previously reported. Cleavage of Os04g44354 and Os03g43930 occurred with higher frequencies at the 9^th^ and 12^th^ positions of miR1435 and miR166, respectively, in all 12 sequenced clones. This is in contrast to the commonly observed 10^th^ or 11^th^ position of miRNAs, such as the cleavage sites of miR444b.2 on Os04g38780, and miR160 on Os04g43910 and Os04g59430. We also observed a putative target, Os10g30150, for the novel miRNA candidate Can_miR_06, where only three of 10 sequenced clones had cleavage sites at the sixth position; the other degraded fragments were not located on the targeted sequence at all (data not shown).

Finally, quantitative real time-PCR (qRT-PCR) was used to examine the correlation of the expression patterns of miRNAs and their targets. Most of the miRNAs were negatively associated with their targets (Additional file 7B). As shown in Table 3, a large number of targets of differentially expressed miRNAs during grain filling were transcription factors (Table 3). Based on their biological functions in rice or other species, the predicted target genes appear to be involved in various bio-logical processes. For example, miR159 regulates a MYB gene, which is considered a positive regulator of the GA response during grain maturation [36]. The targets of miR160, Os04g43910 and Os04g59430, are auxin-responsive factors (ARFs), which are important components of auxin signal transduction [37]. MiR444 targeted a type of MADS-box transcription factor that is similar to an Arabidopsis homolog that has roles in fruit dehiscence [38]. Moreover, transcription factors, such as NAC domain proteins, growth factors, and the SCARECROW gene regulator, have been observed in other cellular growth developmental processes. Differential expression of miRNAs and their target genes seem to form a complicated regulatory network that plays a critical role during grain filling in rice.

**Table 3:** Potential targets of miRNAs with significantly changed expression during rice grain filling

**Name**	**Putative targets (score)**	**Functions**	**Pathway**
**Up-regulated**			
miR159	LOC_Os01g12700(1)	Myb-like DNA-binding domain	Signal transduction or/TF
	LOC_Os05g41166(1)	Transcription factor GAMYB	Signal transduction or/TF
miR164	LOC_Os02g36880(1)	NAC domain protein NAC5	Signal transduction or/TF
	LOC_Os06g46270(1)	NAC domain-containing protein 21/22	Signal transduction or/TF
miR1850	LOC_Os04g33510 (2.5)	Expressed protein	Unknown pathway
	LOC_Os09g29200 (3.5)	Glutathione S-transferase	Redox homeostasis
miR1862	LOC_Os02g30730(3)	SART-1 family protein	Cell cycle/biogenesis
	LOC_Os02g48300(4)	Flavonol synthase/flavanone 3- hydroxylase	secondary metabolism
	LOC_Os05g03174(4)	F-box domain containing protein	Protein degradation
	LOC_Os01g53700(4.5)	Glycosyltransferase	Carbohydrate metabolism
	LOC_Os03g58290(4.5)	Mitochondrial-processing peptidase alpha subunit	Amide acid metabolism
	LOC_Os07g22930(4.5)	Granule bound starch synthase GBSS1b,	Carbohydrate metabolism
	LOC_Os07g09460(4.5)	Indole-3-glycerol phosphate lyase	Lipid metabolism
miR1874-3p	LOC_Os01g05680(4)	Geranylgeranyl transferase type-1 beta subunit	Protein folding and transport
	LOC_Os07g38110(5)	Tic20-like protein	Protein folding and transport
miR1874-5p	LOC_Os03g02590(3.5)	Peroxisomal membrane protein PEX11-	Redox homeostasis
	LOC_Os05g18774(3.5)	Protein phosphatase 1	Signal transduction or/TF
	LOC_Os01g16870(4.5)	Argonaute-like protein	RNA processing
	LOC_Os08g28410(4.5)	Anther-specific proline-rich protein APG	Organ development
	LOC_Os06g51084(5)	1,4-alpha-glucan branching enzyme	Carbohydrate metabolism
**Down-regulated**			
miR160	LOC_Os06g47150(1)	Auxin response factor	Signal transduction or/TF
miR166	LOC_Os03g43930(3.5)	Class III HD-Zip protein 4	Signal transduction or/TF
	LOC_Os03g01890(3.5)	Rolled leaf1	Organ development
miR167	LOC_Os04g57610(3.5)	Auxin response factor 8	Signal transduction or/TF
miR171	LOC_Os02g44360(0)	SCARECROW gene regulator	Signal transduction or/TF
miR396	LOC_Os02g47280(1)	Growth-regulating factor	Cell cycle/biogenesis
miR444	LOC_Os02g36924(0)	MADS-box transcription factor 27	Signal transduction or/TF
	LOC_Os04g38780(0)	MADS-box transcription factor 27	Signal transduction or/TF
	LOC_Os02g49840(1)	MADS-box transcription factor 57	Signal transduction or/TF
	LOC_Os08g06510(2)	Zinc finger, C3HC4 type family protein	Signal transduction or/TF
miR530	LOC_Os02g14990(2)	Zinc finger, C3HC4 type family protein	Signal transduction or/TF
	LOC_Os01g01120(2.5)	E-1 enzyme, putative	Protein degradation
	LOC_Os01g43380(3)	Transferase, transferring glycosyl groups	Carbohydrate metabolism

## Discussion

Using high throughput sequencing and customized miRNA chips, we analyzed small RNAs in developing rice grains from the milky to hard dough stages. The analysis revealed dynamic features of the regulatory network mediated by miRNAs during rice grain development.

### Small RNA population and novel miRNAs involved in developing grains

We obtained nearly 2 million high quality small RNAs from grain samples collected from 6 to 20 DAF. A significant proportion of the small RNAs were 21 nt to 24 nt in length. In plants, 21-nt miRNAs and trans-acting siRNAs have roles in post-transcriptional gene silencing by directing mRNA degradation or translational repression [1,39], whereas 24-nt siRNAs tend to be involved in DNA and histone modifications that lead to transcriptional gene silencing [40,41]. Recently, 24-nt miRNAs were also found to direct DNA methylation [42]. In our sequencing data, the reads of 24-nt small RNAs were nearly 7-fold more frequent than reads for 21-nt small RNAs (Additional file 2). The presence of a large population of small RNAs in developing rice grains suggests they have important roles in transcriptional and post-transcriptional regulation of the genes involved in grain development.

Although a number of studies of small RNAs have been carried out using grains from various developmental stages and from various rice accessions, novel miRNAs involved in this process have been continuously discovered [16,26,27]. We sequenced small RNA pools from the developing caryopsis of the indica landrace, Baifeng B, at different stages of development and revealed many classes of conserved miRNAs as well as novel ones. The discovery of 11 novel miRNA candidates was supported by detection of corresponding miRNA*s that were consistent with recent miRNA criteria for characterization [29]. No homologous members were reported in other species, indicating that they are probably rice-specific and found only with extensive tissue sampling.

### miRNAs have dynamic expression patterns in developing grains

Many miRNAs display temporal or tissue-specific expression patterns [15,43-45]. Our sequencing results revealed that more than 100 known rice miRNAs were expressed in the rice grain. Several, such as miR156, miR159, miR164, miR166, miR167 and miR396, were expressed at high levels, indicating that, as they are highly expressed in other tissues such as leaf and root, these conserved miRNAs are possibly important regulators for rice plant development.

Our chip data showed that known and novel miRNAs were expressed differentially during the grain filling process. Approximately half of the conserved miRNAs detected were up-regulated from 6 to 20 DAF, whereas approximately half were down-regulated. Compared with previous reports [28], the expression levels of most miRNAs were approximately the same or up-regulated during the periods 1–5 and 6–10 DAF. Some miRNA genes, such as miR159 and miR399, displayed continually high expression levels throughout grain filling. In contrast, the expression levels of miR160, miR166, miR167, miR171, miR396 and miR444 were down-regulated at the late phase after being up-regulated during early development (Additional file 8). Previous work on gene expression showed that in the early development phase (6–12 DAF) grain metabolic pathways tend to involve embryo differentiation and cell enlargement [24,46]. This pattern changes at the soft-dough stage (13–17 DAF) and during the late filling phase when grains begin to lose moisture and metabolism switches to senescence and dormancy, processes that might be associated with down-regulated patterns of some miRNAs.

### A complex regulatory network in rice grain development

Our results showed that differentially expressed miRNAs seem to regulate large numbers of genes, including many transcription factor genes. In previous microarray analyses, a group of transcription factor genes identified to be involved in the transcriptional control of grain filling included a ZIP type transcription factor that was highly expressed in aleurone and endosperm, and certain MYB genes that may be important in regulating gene expression in developing rice grains [24,47]. On the other hand, NAC domain protein genes regulated by miR164 were implicated in regulating metal mobilization from leaves to seed [48], as well as grain senescence and nutrient remobilization [49], while MADS box transcript genes, the targets of miR444, were considered necessary for fruit ripening in tomato and embryo development in Arabidopsis [16,38,50,51]. In addition, hormonal accumulation and other changes in seeds were shown to affect nitrogen supply and drought tolerance during grain filling [52]; for example, miR160 targets ARFs that can bind auxin-response elements to regulate expression of other genes [37]. Novel miRNAs are often expressed at low levels and match their targets with imperfect pairing. We propose that novel miRNAs may be involved in rice grain development by targeting starch synthesis genes that control the accumulation of starch. Although we were unable to identify the exact cleavage sites on the targets, these novel miRNAs probably regulate their targets by translational inhibition. In light of their important functions in the regulatory network of grain development, future work on these miRNAs and their targets is required.

## Conclusions

This work provides the first small RNA expression analysis throughout the entire grain filling phase in an indica rice cultivar. Our small RNA sequencing and chip analysis enlarged the rice miRNA repertoire and confirmed the existence of most conserved, and nearly half of the non-conserved, rice miRNAs in developing grains. Comparison between the three phases of grain filling revealed that these miRNAs and their targets may be involved in diverse pathways, which may also be conserved in other cereal plants.

## Methods

### Plant materials and construction of a small RNA library

Baifeng B (an indica landrace) was grown under normal field conditions. Immature grains (and husks) were collected at different developmental stages: milk-ripe (5–12 DAF), soft-dough (13–17 DAF) and hard-dough (18–20 DAF). Total RNAs were extracted and equally mixed to construct a library. Small RNAs of 17–27 nt were separated and purified by denaturing polyacrylamide gel electrophoresis. After dephosphorylation and ligation to an adapter, the products were reverse-transcribed and amplified by PCR, and were later sequenced using Illumina technology.

### Bioinformatics analysis and target validation

Primers and 3′/5′ adaptors were removed from the original reads and other contaminants (rRNA, tRNA, snRNA and snoRNA) were removed using RepeatMasker. Small RNA sequences of 18 to 26 nt were collected and subjected to BLAST analysis against the *Oryza sativa* ssp. indica 9311 sequence (TIGR Rice Annotation Release 5.0) using SOAP aligner (−s 8 -v 0 -r 2). Whole matching sequences were compared with annotated rice miRNAs and their precursors in miRBase (version 16.0, http://www.mirbase.org); homologs of the indica 93–11 genome were regarded as mature miRNAs and miRNA precursors based on Patscan searches (mismatch < =2, insertion = 0, deletion = 0). MiRNAs located at the position ±2 nt of the precursors were also included as mature miRNAs. New miRNA prediction was based on the rules described by Sunkar et al. [26]. We ran Mfold software using Perl script to identify novel miRNAs; we used a 20 bp frame to search sequences 20 to 260 bp upstream and downstream of each miRNA. Candidate miRNA identification standards were those suggested by Meyers et al. [31], miRNA/miRNA* region with < = 3 bulges, total mismatches < = 6 bases [53]. Candidate targets were identified by miRU following methods previously described [54].

### Gene expression analysis using microarray hybridization

Grain samples were collected at three stages: milk-ripe (6–12 DAF), soft-dough (13–17 DAF) and hard-dough (18–20 DAF) with three biological replicates for each stage. Total RNA (5 μg) was used as the starting material for each assay. RNAs were size-fractionated using a YM-100 Microcon centrifugal filter (Millipore, Bedford, MA, USA), and the small RNAs (300 nt) were isolated and extended with a poly(A) tail using poly(A) polymerase. miRNA microarray chips were fabricated by LC Sciences, Houston, Texas, USA. A total of 546 probes were spotted on each chip, including 254 known miRNAs from miRBase version 13.0, 11 newly identified candidates and 50 controls with six duplications. Rice 5 S rRNA served as an inner positive control; and PUC2-20B, an artificial non-homologous nucleic acid, was used as an external positive control. Perfect match and single-base mismatch counterparts to the external positive control, named PUC2PM-20B and PUC2MM-20B, were spiked into the RNA samples before probe labeling. Blank and non-homologous nucleic acids were used as negative controls. Chip hybridization experiments were carried out in triplicate using different biological samples. Hybridization images were collected using a laser scanner (GenePix 4000B, Molecular Devices, Sunnyvale, CA, USA) and digitized using Array-Pro image analysis software (Media Cybernetics, Silver Spring, MD, USA). Signal values were derived by background subtraction and normalization. A transcript to be listed as detectable had to fulfill at least two conditions: signal intensity higher than 3× (background standard deviation) and spot coefficient of variation (CV) < 0.5. CV was calculated by (standard deviation)/(signal intensity). When repeating probes were present on an array, a transcript was listed as detectable only if the signals from at least 50% of the repeating probes were above detection level [55]. Student’s t-tests were used to assess differences in miRNA levels. To minimize noise and improve accuracy, some probes detected with low abundance (signal value <100) were not included in variance analysis. Signals below the background average (signal value <32) were considered non-expressing (Additional file 3).

### Northern blot analysis

Low molecular weight RNA (30 μg) was loaded per lane, resolved on a 15% denaturing polyacrylamide gel, and transferred electrophoretically to Hybond-N^+^ membranes (Amersham Biosciences, UK). Both sides of membranes were UV cross-linked for 2 minutes and baked for 1 h at 80°C. DNA oligonucleotides complementary to miRNA sequences were end-labeled with r-^32^P-ATP using T4 polynucleotide kinase (NEB, Beijing). Membranes were hybridized in hybridization buffer (Ambion, US) for 16 h at 42°C. Blots were washed three times with 1× saline sodium citrate and 0.5% sodium dodecyl sulfate at 42°C. Membranes were briefly air-dried and wrapped with Saran Wrap. Images were acquired using a Molecular Imager FX instrument (BioRad, USA).

### RNA ligase-mediated 5′ RACE and quantitative RT-PCR

Total RNA from rice grain samples that combined equal amounts of material collected at the milk ripe, soft dough and hard dough stages was used to construct a 5′-RACE library. We used the PolyATract mRNA isolation system (Promega) and the GeneRacer kit (Invitrogen) according to the manufacturer’s instructions. Two outer and inner-specific primers were used for each RACE reaction (Additional file 9). Amplicons were separated by agarose electrophoresis, cloned into pMD 19-T (Takara) and sequenced. A minimum of six clones were sequenced for each PCR product. In the quantitative RT-PCR experiments of mRNAs, total RNAs were reverse-transcribed using poly(T) adapter. SYBR® Green PCR Master Mix (Takara) was used in all quantitative RT-PCR experiments. The relative fold expression changes of target genes were calculated using the 2 delta-delta Ct method. Primers used in all quantitative RT-PCR experiments are listed in Additional file 10.

## Competing interests

The authors declare that they have no competing interests.

## Authors’ contributions

YL, NS and JMW designed the experiment. ZJC and JLW supplied the experimental material. YL and YS collected the samples and carried out the laboratory analyses. YL, RZZ and FQW analyzed the data. YL, LM, and JMW wrote the manuscript. LJ, XPG, JW and JMW supervised the laboratory work. All authors have read and approved the final manuscript.

## Supplementary Material

Additional file 1 Data quality of sRNA library. Click here for file

Additional file 2 Size distribution of small RNAs. Click here for file

Additional file 3 A The abundance of known miRNAs in the library-1. Click here for file

Additional file 4 Secondary structures of novel miRNAs. Click here for file

Additional file 5 Predicted targets of novel miRNAs. Click here for file

Additional file 6 A Expression patterns of conserved miRNAs. Click here for file

Additional file 7 Validation and expression of selected miRNA target genes. Click here for file

Additional file 8 miRNA variant patterns among grain developing stages. Click here for file

Additional file 9 5′RLM-RACE primers used in this study. Click here for file

Additional file 10 Quantitative RT-PCR primers. Click here for file
