# Supplementary material for: Identification of novel MiRNAs and MiRNA expression profiling during grain development in indica rice
Source: BMC Genomics. 2012 Jun 21;13:264. doi: 10.1186/1471-2164-13-264 (PMC3505464; doi:10.1186/1471-2164-13-264)
Supplement: Additional file 1 — Data quality of sRNA library. [file 1471-2164-13-264-S1.doc]

## Additional file 1. Data quality of sRNA library.

|  | Total (%) | Unique (%) |
| --- | --- | --- |
| Sequencing reads | 1832288 (%) | 974934 (%) |
| Singleton Reads | 824768 (45%) | 824768 (84.6%) |
| Perfect match to 9311 genome | 1363246 (74.4%) | 637362(65.4%) |
| Match known miRNA reads | 146498 (8%) | 140 (0.01%) |

Numbers in brackets indicate the percentage of small RNAs of total or unique signatures.
