# Supplementary material for: Identification of novel MiRNAs and MiRNA expression profiling during grain development in indica rice
Source: BMC Genomics. 2012 Jun 21;13:264. doi: 10.1186/1471-2164-13-264 (PMC3505464; doi:10.1186/1471-2164-13-264)
Supplement: Additional file 2 — Size distribution of small RNAs. [file 1471-2164-13-264-S2.doc]

A. Size distribution of small RNA unique reads

B. Size distribution of small RNA raw reads

### Additional file 2. Size distribution of small RNAs
