# Supplementary material for: Identification of novel MiRNAs and MiRNA expression profiling during grain development in indica rice
Source: BMC Genomics. 2012 Jun 21;13:264. doi: 10.1186/1471-2164-13-264 (PMC3505464; doi:10.1186/1471-2164-13-264)
Supplement: Additional file 3 — A The abundance of known miRNAs in the library-1. [file 1471-2164-13-264-S3.pdf]

Additional file 3A The abundance of known miRNAs in the library-1

Family	miRNA ID	Reported sequence	miRNA abundance	Variants sequence *	Locus	Chr	miRNA*	miRNA* abundance
**conserved**								
MIR156	osa-miR156a-j	UGACAGAAGAGAGUGAGCAC	538		a	1		
					b	1		
					c	1		
					d	2	GCUCACUCCUCUUUCUGUCACC	3
					e	4	GCUCACUGCUCUUUCUGUCAUC	1
					f	8		
					g	9		
					h	6	UGCUCGCUCCUCUUUCUGUCAG	6
					i	2	UGCUCACUGCUCUGUCUGUCAUC	1
					j	-	UGCUCGCUCCUCUUUCUGUCAG	6
	osa-miR156k	UGACAGAAGAGAGAGAGCACA	4	UGACAGAAGAGAGAGAGCAC	k	9		
	osa-miR156l	CGACAGAAGAGAGUGAGCAUA	3	CGACAGAAGAGAGUGAGCAU	l	5		
MIR159	osa-miR159a.1	UUUGGAUUGAAGGGAGCUCUG	207		a	1		
	osa-miR159a.2	UUUGGAUUGAAGGGAGCU	7113		b	1		
	osa-miR159c	AUUGGAUUGAAGGGAGCUCCA	9	UUGGAUUGAAGGGAGCUC	c	1		
	osa-miR159d	AUUGGAUUGAAGGGAGCUCCG	9	UUGGAUUGAAGGGAGCUC	d	1		
	osa-miR159e	AUUGGAUUGAAGGGAGCUCCU	9	UUGGAUUGAAGGGAGCUC	e	1		
	osa-miR159f	CUUGGAUUGAAGGGAGCUCUA	19	CUUGGAUUGAAGGGAGCU	f	1	AGCUCCCUUCGAUCCAAUCCA	1
MIR160	osa-miR160a-d	UGCCUGGCUCCCUGUAUGCCA	162		a	2	GCGUGCAAGGAGCCAAGCAUG	2
					b	6	GCGUGCAAGGAGCCAAGCAUG	2
					c	8		
					d	3		
	osa-miR160e	UGCCUGGCUCCCUGUAUGCCG	272		e	7	GCGUGCGAGGUGCCAAGCAUGG	4
	osa-miR160f	UGCCUGGCUCCCUGAAUGCCA	30		f	8	UUGAGGGAGUCAUGCAGGGUU	6
MIR162	osa-miR162a	UCGAUAAACCUCUGCAUCCAG	103		a	2		
	osa-miR162b	UCGAUAAGCCUCUGCAUCCAG	95		b	4	GGGCGCAGUGGUUUAUCGAUC	8
MIR164	osa-miR164a,b,f	UGGAGAAGCAGGGCACGUGCA	6871		a	7		

					b	5	AUGGUUCUUCAUGUGCCCGUC	6
					f	5	UGCAUGUGCCCUUCUUCUCCA	1
	osa-miR164c	UGGAGAAGCAGGGUACGUGCA	24		c	5		
	osa-miR164d	UGGAGAAGCAGGGCACGUGCU	220		d	2		
	osa-miR164e	UGGAGAAGCAGGGCACGUGAG	12	UGGAGAAGCAGGGCACGUG	e	3		
MIR166	osa-miR166a-d,f,	UCGGACCAGGCUUCAUUCCCC	14397		a	10	GGAAUGUUGUCUGGUUCAAGG	1
					b	6	GGAAUGUUGUCUGGCUCGGGG	108
					c	3	UGAGGGGAAUGUUGUCUGGUC	4
					d	2	GGAAUGUUGUCUGGCUCGAGG	48
					f	10		
					n	3		
	osa-miR166e	UCGAACCAGGCUUCAUUCCCC	1		e	3	GGAAUGUUGUCUGGUUCAAGG	1
	osa-miR166g-h	UCGGACCAGGCUUCAUUCCUC	11597		g	12		
					h	2	GGAAUGUUGGCUGGCUCGAGGU	4
	osa-miR166i-j	UCGGAUCAGGCUUCAUUCCUC	464		i	3		
					j	-		
	osa-miR166k-l	UCGGACCAGGCUUCAAUCCCU	1929		k	2	GGUUUGUUGUCUGGCUCGAGGC	7
					l	9	UUGUUGUCUGGUUCAAGGUCU	2
	osa-miR166m	UCGGACCAGGCUUCAUUCCCU	135(482)	UCGGACCAGGCUUCAUUCC	m	8		
MIR167	osa-miR167a-c	UGAAGCUGCCAGCAUGAUCUA	14759		a	12	GAUCAUGCAUGACAGCCUCAUU	1
					b	3		
					c	3		
	osa-miR167d-j	UGAAGCUGCCAGCAUGAUCUG	6473(7656)	TGAAGCTGCCAGCATGATCTGA	d	7		
					e	2	UCAGAUCAUGUUGCAGCUUCA	1
					f	10		
					g	3	UCAUCCGGCAGCUUCAUCUUC	2
					h	12		
					i	6	UCAGAUCAUGUUGCAGCUUCA	1
					j	1	AGAUCGUGCUGCGCAGUUUCAUC	10
MIR168	osa-miR168a	UCGCUUGGUGCAGAUCGGGAC	17200		a	2	GAUCCCGCCUUGCACCAAGUGAAU	4

	osa-miR168b	AGGCUUGGUGCAGCUCGGGAA			b			
MIR169	osa-miR169a	CAGCCAAGGAUGACUUGCCGA	18		a	1	CUACAUCUUGGCCCCUGCUCC	10
	osa-miR169b-c	CAGCCAAGGAUGACUUGCCGG	32		b	6		
					c	4		
	osa-miR169d	UAGCCAAGGAUGAAUUGCCGG	4		d	9		
	osa-miR169e	UAGCCAAGGAUGACUUGCCGG	15		e	2		
	osa-miR169f-g	UAGCCAAGGAUGACUUGCCUA			f	2		
					g	4		
	osa-miR169h-m	UAGCCAAGGAUGACUUGCCUG	1		h	8		
					i	8		
					j	9		
					k	9		
					l	8		
					m	8		
	osa-miR169n-o	UAGCCAAGAAUGACUUGCCUA	2		n	11		
					o	11		
	osa-miR169p	UAGCCAAGGACAAACUUGCCGG			p	8	CCGGCAAGUCAUCCUUGGCUG	32
	osa-miR169q	UAGCCAAGGAGACUGCCCAUG			q	8	CAGGCAAGUCAUCCUUGGCUA	1
MIR171	osa-miR171a	UGAUUGAGCCGCGCCAAUAUC	12	UUGAGCCGCGCCAAUAUCUCU	a	6		
	osa-miR171b-f	UGAUUGAGCCGUGCCAAUAUC	153		b	8	ACGGGAUAUUGGGGCGGUUCAAUC	4
					c	4	ACGGGAUAUUGGUGCGGUUCA	3
					d	10	UGUUGGCCCGGCUCACUCAGA	19
					e	3	UGUUGGCUCGGCUCACUCAGA	3
					f	3		
	osa-miR171g	GAGGUGAGCCGAGCCAAUAUC			g	7		
	osa-miR171h	GUGAGCCGAACCAAUAUCACU			h	4		
	osa-miR171i	GGAUUGAGCCGCGUCAAUAUC	13	UUGAGCCGCGUCAAUAUCUC	i	3	GGUAUUGGCGUGCCUCAAUC	1
MIR172	osa-miR172a,d	AGAAUCUUGAUGAUGCUGCAU	267		a	9		
					d	2		
	osa-miR172b	GGAAUCUUGAUGAUGCUGCAU	38		b	1		

	osa-miR172c	UGAAUCUUGAUGAUGCUGCAC			c	2		
MIR159	osa-miR319a-b	UUGGACUGAAGGGUGCUCCC	1(27)	UUGGACUGAAGGGUGCUCCCU	a	1		
					b	1	AAGAGAGCGUCCUUCAGUCCACUC	1
MIR390	osa-miR390	AAGCUCAGGAGGGAUAGCGCC	44			3	AGGCGCUAUCUAUCCUGAGCUCCA	3
MIR393	osa-miR393	UCCAAAGGGAUCGCAUUGAUC	15			1		
	osa-miR393b	UCCAAAGGGAUCGCAUUGAUCU	15	UCCAAAGGGAUCGCAUUGAUC	b	4	UCAGUGCAAUCCCUUUGGAAU	16
MIR394	osa-miR394	UUGGCAUUCUGUCCACCUCC	89			2		
MIR395	osa-miR395a	GUGAAGUGCUUGGGGGAACUC			a	4		
	osa-miR395b,d-e	GUGAAGUGUUUGGGGGAACUC			b	4		
			1	ACUAUGUGAAGUGUUUGGGGGA	d	4		
					e	4		
					g	4		
					h	8		
					i	8		
					j	8		
					k	8		
					l	8		
					m	4		
					n	4		
					p	4		
					q	4		
			1	ACUAUGUGAAGUGUUUGGGGGA	r	4		
					s	4	CCGUGAGUUCCCUUCAAGCACU	1
	osa-miR395c	GUGAAGUGUUUGGAGGAACUC			c	4		
	osa-miR395f	GUGAAUUGUUUGGGGGAACUC			f	4		
	osa-miR395o	AUGAAGUGUUUGGAGGAACUC			o	4		
	osa-miR395t	GUGAAGUGUUUGGGGAAACUC			t	9		
	osa-miR395u	GUGAAGCGUUUGGGGGAAAUC			u	9		
	osa-miR395v	GUGAAGUAUUUGGCGGAACUC			v	9		
	osa-miR395w	GUGAAGUGUUUGGGGGAUUCUC			w	9		

MIR396	osa-miR396a-b	UUCCACAGCUUUCUUGAACUG	16		a	2	GUUCAAUAAAGCUGUGGGAAA	2
					b	6	GUUCAAUAAAGCUGUGGGAAA	2
	osa-miR396c	UUCCACAGCUUUCUUGAACUU	9		c	2	GGUCAAGAAAGCUGUGGGAAG	9
	osa-miR396d-e	UCCACAGGCUUUCUUGAACUG	230		d	4		
					e	-	GUUCAAGAAAGCCCAUGGAAA	1
	osa-miR396f	UCUCCACAGGCUUUCUUGAACU	230	UCCACAGGCUUUCUUGAACUG	f	2	GUUCAAGAAAGUCCUUGGAAA	7
	osa-miR396g-i	UCCACAGGCUUUCUUGAACGG	16	UCCACAGGCUUUCUUGAA	g	6		
			16	UCCACAGGCUUUCUUGAA	h	2		
			16	UCCACAGGCUUUCUUGAA	i	4		
MIR397	osa-miR397a	UCAUUGAGUGCAGCGUUGAUG	38(40)	AUUGAGUGCAGCGUUGAUGAA	a	6		
	osa-miR397b	UUAUUGAGUGCAGCGUUGAUG	93	UUGAGUGCAGCGUUGAUGAACC	b	2	UCACCAGCACUGCACCCAAUC	29
MIR398	osa-miR398a	UGUGUUCUCAGGUCACCCCUU			a	10		
	osa-miR398b	UGUGUUCUCAGGUCGCCCCUG	6		b	7		
MIR399	osa-miR399a-c	UGCCAAAGGAGAAUUGCCCUG			a	1		
					b	2		
					c	5		
	osa-miR399d	UGCCAAAGGAGAGUUGCCCUG	2		d	6		
	osa-miR399e-g	UGCCAAAGGAGAUUUGCCCAG			e	1		
					f	6		
					g	2		
	osa-miR399h	UGCCAAAGGAGACUUGCCCAG			h	5		
	osa-miR399i	UGCCAAAGGAGAGCUGCCCUG			i	2		
	osa-miR399j	UGCCAAAGGAGAGUUGCCCUA	2		j	4		
	osa-miR399k	UGCCAAAGGAAAUUUGCCCCG			k	5		
MIR408	osa-miR408	CUGCACUGCCUCUUCCCUGGC	104			1	ACAGGGAUGAGGCAGAGCAUG	28
**unconserved**								
MIR413	osa-miR413	CUAGUUUCACUUGUUCUGCAC				12		
MIR414	osa-miR414	UCAUCCUCAUCAUCAUCGUCC				11		
MIR415	osa-miR415	AACAGAACAGAAGCAGAGCAG				12		
MIR416	osa-miR416	UGUUCGUCCGUACACUGUUCA				4		

MIR417	osa-miR417	GAAUGUAGUGAAUUUGUUCCA				8		
MIR418	osa-miR418	UAAUGUGAUGAUGAAAUGACG				2		
MIR419	osa-miR419	UGAUGAAUGCUGACGAUGUUG				12		
MIR420	osa-miR420	UAAAUUAAUCACGGAAAUGAU				4		
MIR426	osa-miR426	UUUUGGAAGUUUGUCCUUACG				5		
MIR435	osa-miR435	UUAUCCGGUAUUGGAGUUGA				3		
MIR437	osa-miR437	AAAGUUAGAGAAGUUUGACUU				2		
MIR438	osa-miR438	UUCCCACGCGUUAUAGUGAAA				6		
MIR439	$osa-miR439a-j	UGUCGAACCGCGGUUGUUCGA	11	GGUAUCCCUGUCGAACCGCGGUU	a	1	ACUGACGCAGUUCGACAUGUACCU[illegible]	1
			2	UCGAACCGCGGUUGUUCGACA	b	10		
					c	7		1
					d	3		
					e	8		
					f	9		
					g	8		
					h	6		
					i	-		
					j	10		
MIR440	osa-miR440	AGUGUCUCCUGAUGAUCGGGACAA				11		
MIR441	osa-miR441a-c	UACCAUCAAUAUAAAUGUGGGAAA			a	3		
		441b-5p			b	7		
					c	1		
MIR445	osa-miR442	UGACGUGUAAAUUGCGAGACGAAU				4		
MIR443	osa-miR443	AUCACAAUACAAUAAAUCUGGA				3		
MIR444	osa-miR444a.1,d	UUGCUGCCUCAAGCUUGCUGC	1		a	-		
					d	-		
	osa-miR444a.2,d	UGCAGUUGCUGCCUCAAGCUU	295		a	-	GCUAGAGGUGGCAACUGCAUA	3
					d	-		
					e	-		
	osa-miR444b.1,c	UGUUGUCUCAAGCUUGCUGCC	153		b	-		

					c	-		
	osa-miR444b.2,c	UGCAGUUGUUGUCUCAAGCUU	3917		b	-	AGAGACAGCAACUGCAUAUCU	5
					c	-		
	osa-miR444d.3	UUGUGGCUUUCUUGCAAGUUG	8		d	-		
	osa-miR444f	UGCAGUUGUUGCCUCAAGCUU	178		f	-		
MIR445	$osa-miR445a-i	UAAAUUAGUGUAUAAACAUCCGA	4	UUUAAGCCUAAUUAAUCCAUAA	a	7		
					b	-		
					c	-		
					d	5		
					e	-		
					f	-		
					g	-		
					h	-		
					i	-		
MIR441	osa-miR446*	CAUCAAUAUGAAUAUGGGAAAUG	1	ACAUCAAUAUGAAUAUGGGAAAUG		6		
			7	ACUUACAUUGUGAAACGGAGGGAG				
MIR528	osa-miR528	UGGAAGGGGCAUGCAGAGGAG	6305			3		
MIR529	osa-miR529a	CUGUACCCUCUCUCUUCUUC			a	2	AGAAGAGAGAGAGUACAGCCU	135
	osa-miR529b	AGAAGAGAGAGAGUACAGCUU		*AGAAGAGAGAGAGTACAGCCT*	b	4		
MIR530	osa-miR530-3p	AGGUGCAGAGGCAGAUGCAAC	2			4		
	osa-miR530-5p	UGCAUUUGCACCUGCACCUA	22	UGCAUUUGCACCUGCACCUAC		4		
MIR531	osa-miR531	CUCGCCGGGGCUGCGUGCCGCCAU				8		
	osa-miR531b	CUCGCCGGGGCUGCGUGCCG	2	CUCGCCGGGGCUGCGUGCCGC	b	1		
MIR535	osa-miR535	UGACAACGAGAGAGAGCACGC	1918			11	UGCUUUCUCCCGUUGUCACUG	16
MIR806	osa-miR806a-h	AUGUGCUAAAAAGUCAACGGUG	1(22)	CCGUUUCAAAAUGUUUGACACC	a	1		
					b	2		
			17	AACAUGUGCUAAAAAGUCAACG	c	3		
			7	AUGUGCUAAAAAGUCAACGGUG	d	4		
					e	7		
					f	8		

					g	10		
					h	11		
MIR807	osa-miR807a-c	CGUCAUCUCACAGGUGAAUCC			a	1	CAAUGACAUGCAUCCUAUUACCAG	1
					b	2		
					c	4	AUGGAUUCACCCGUGAUAUGACGU	2
MIR441	osa-miR808	AUGAAUGUGGGAAAUGUAAGAA	1	AUCAAUAUGAAUGUGGGAAAU		3		
MIR441	osa-miR809a-h	UGAAUGUGAGAAAUGUUAGAAU	1	UUAGAAUGACUUACAUUGUGA	a	3	UCCGUUUCACAAUGUAAGUCA	6
			76	AGAAUGACUUACAUUGUGAAAC	b	5		
			2	AAAUGUUAGAAUGACUUACAUU	c	6	UCACAAUAUAAGUCAUUCUAGCAU	3
					d	7		
					e	8		
					f	9		
					g	11		
					h	12		
MIR810	osa-miR810	UCAUAAGCCCACCACAUGUGG			a	-		
		UGAACACCGAUAUGCGUCAUC			b	11		
MIR811	osa-miR811a-c	ACCGUUAGAUCGAGAAAUGGACGU		CUACCGUUAGAUCGAGAAAUGG	a	6		
					b	10		
					c	11		
MIR812	osa-miR812a-e	GACGGACGGUUAAACGUUGGAC	3	AAGACGGACGGUUAAACGUUGG	a	1		
					b	2		
					c	6		
					d	7		
					e	8		
	osa-miR812f				f	4		
	osa-miR812g-j	AAGACGGAUGAUUAAAGUUGGAC	1(4)	AAUAAGACGGAUGAUUAAAGUU	g	3		
					h	3		
					i	7		
					j	8		
MIR445	osa-miR813	GGGUUAUGGAAUGGGUUUUACC	1	AAUAGCCUAGGGGUUAUGGAAUGG		11		

MIR814	osa-miR814a-c	CACUUCAUAGUACAACGAAUCU	2	ACGAGACACUUCAUAGUACAACC	a	1	
					b	2	
					c	2	
MIR815	osa-miR815a-c	AAGGGGAUUGAGGAGAUUGGG	1(4)	AAGGGGAUUGAGGAGAUUGGGG	a	5	
			15	AAGGGGAUUGAGGAGAUUGGGA	b	5	
					c	7	UCCUCUCUUCUCUCCUCCACCUCA
	osa-miR816	GUGACAUAUUUUACUACAAC	1	UUUUACUACAACGAAUCUGGAUAU		10	
	osa-miR817	UCCAACUUGAGGCCCGAUUGA				2	
MIR812	osa-miR818a-e	AAUCCCUUAUAUUAUGGGACGG	1	AAAAUCCCUUAUAUUAUGGGACG	a	1	
					b	2	
					c	2	
					d	4	
					e	7	
MIR441	osa-miR819a-k	UCAGGUUAUAAGACUUUCUAGC	162	CCUCCGUUUCAGGUUAUAAGACU	a	1	
					b	2	
					c	2	
					d	3	
					e	3	
					f	4	
					g	5	
					h	6	
					i	7	
					j	8	
					k	10	
MIR820	osa-miR820a-c	UCGGCCUCGUGGAUGGACCAG	10(415)	UCGGCCUCGUGGAUGGACCAGGA	a	1	
				TCGGCCTCGTGGATGGACCAGGA			
					b	7	
					c	10	
MIR821	osa-miR821a-c	AAGUCAUCAACAAAAAAGUUGAAU			a	3	
					b	7	

			1	AUACGACAAAGUGAUAGUAACAU	c	8	
	osa-miR827	UAAGAUGACCAUCAGCGAAAA				8	
MIR827	osa-miR827a-b	UUAGAUGACCAUCAGCAAACA			a	2	UUUUGUUGCUGGUCAUCUAG
					b	-	
MIR1882	osa-miR1317-5p.	AGAUUGCUUUCAAGGUCAUUUCUU				10	
	osa-miR1317-5p	UAGGGAACCCCAUUCCCAUAAA					
	osa-miR1317-3p	GAAAUGAUCUUGGACGUAAUCUAG					
MIR1318	osa-miR1318	UCAGGAGAGAUGACACCGAC	1	AUCAGGAGAGAUGACACCGACA		7	
	osa-miR1319	AACCGGCAUCUGUAAUAUAUUAU	1	AAAACCGGCATCTGTAATATATTA		11	
	osa-miR1320	UGGAACGGAGGAAUUUUAUAG				6	
MIR1423	osa-miR1423-5p.	AGGCAACUACACGUUGGGCGCUC	4			4	
	osa-miR1423b	CAACUACACGUUGGGCGCUCGA	28		b	-	
	osa-miR1424	AUGCACACUGAUGCUGAUUGU				7	
	osa-miR1425	UAGGAUUCAAUCCUUGCUGCU				5	
	osa-miR1426	AGAAUCUUGAUGAUGAUUAAA				1	
	osa-miR1427	UGCGGAACCGUGCGGUGGCGC	1			8	
MIR1428	osa-miR1428a-3p	UAAGAUAAAGCCGUGAAUUUG				1	
	osa-miR1428a-5p	CGUUUUGCAAAUUCGCAGGCC				1	
	osa-miR1428b-d	UAAGAUAAUGCCAUGAAUUCG			b	1	
					c	2	
					d	3	
	osa-miR1428e-3p	UAAGAUAAUGCCAUGAAUUUG				3	
	osa-miR1428e-5p	AAUUCACAGGCCCUAUCUUGUG				3	
	osa-miR1428f-5p	AAUUCACAGGCCCUAUCUUGUG				7	
	osa-miR1428g-5p	AAUUCACAGGCCCUAUCUUGUG				10	
	osa-miR1429-3p	GUUGCACGGGUUUGUAUGUUG	1(2)	GUUGCACGGGUUUGUAUGUUGC		8	
	osa-miR1429-5p	GUAAUAUACUAAUCCGUGCAU	1	GTAATATACTAATCCGTGCATCCA		8	
MIR169_7	osa-miR1430	UGGUGAGCCUUCCUGGCUAAG				12	
	osa-miR1431	UUUGCGAGUUGGCCCGCUUGC				1	
MIR1318	osa-miR1432	AUCAGGAGAGAUGACACCGAC	1	AUCAGGAGAGAUGACACCGACA		-	

MIR169_1	osa-miR1433	UGGCAAGUCUCCUCGGCUACC				3	UAGCCAAGGAUGAUUUGCCUGU	
MIR1435	osa-miR1435	UUUCUUAAGUCAAACUUUUU				1		
MIR818	osa-miR1436	ACAUUAUGGGACGGAGGGAGU	1			1	UACUCCCUCCGUCCCAUAAUAU	1(11)
	osa-miR1437	UCCGGCGCCGCACUAGGCACUG				8	GAGGGAGGGAACGGUGCCUAGU	1
	osa-miR1438	AGGGUAAUUUUAUCAUUUUUAA				10		
MIR818	osa-miR1439	UUUUGGAACGGAGUGAGUAUU		AAAAAUGCUUAUAUUUUGGA		12		
	osa-miR1440	UGCUCAAAUACCACUCUCCU	1	UGCCAAUGCUCAAAUACCACUCUC		9		
	osa-miR1441	ACCGGAUGUCGGAAAAGGUUU	16	UUUUGGGUGUCACAUCGGACGUUU		12		
MIR818	osa-miR1442	AUUCAUAGUACUAGAUGUGU	2	AUGUGUCACAUCCAGUACUAGGUU		7		
MIR1846	osa-miR1846a-3p	UGACCCCGUUCUCCUCGCCGG	1		a	10		
					b	11		
	osa-miR1846a-5p	AGUGAGGAGGCCGGGGCCGCU			a	10		
					b	11		
	osa-miR1846c-3p	UGACCCCGGUCUGCUCGCUGG				12		
	osa-miR1846c-5p	AGUGAGGAGGCCGGGGCCGCU				12		
	osa-miR1846d-3p	UAUCCGGCGCCGCAGGGAGG				1		
	osa-miR1846d-5p	UCCCACCGAGCAGCCGGAUCUC	4			1		
	osa-miR1846e	CAACGAGGAGGCCGGGACCA	1	AACGAGGAGGCCGGGACCACCGGA		9		
	osa-miR1847.1	UGCAGUUUGCAGUUGUGGCAC	1	UUUGUGCAGUUUGCAGUUGUG		1		
	osa-miR1847.2	UGGCCCACAUGUUAGUGCCACAAC				1		
	osa-miR1848	CCUCGCCGGCGCGCGCGUGCA	1	UGCGCGCGGCCGUCGAGUGG		4		
	osa-miR1849	UAUCGUAUCCUAGGUUGGUUU	2(15)	AAUGCCCUAUCGUAUCCUAGGUUG		4	GCUAACCUAGGAUAUGAUGGGACA	1
	osa-miR1850.1	UGGAAAGUUGGGAGAUUGGGG	33(48)	UGGAAAGUUGGGAGAUUGGG		5		
	osa-miR1850.2	UUGUGUGUGAACUAAACGUGG				5		
	osa-miR1850.3	CUGUUUAGUUCACAUCAAUCUU				5		
	osa-miR1851	CGUCUGGGAUGGCAUUUUGGC		GCCGCCGGCGAGCUCUUCACG		5		
	osa-miR1852	AUAUGGAUUCAGAAUGCAGGU				5		
	osa-miR1853-3p	UAAUUGGGGAUGUUCGGUUGCU	1	AAUGGUAAUUGGGGAUGUUCGGUU		8		
	osa-miR1853-5p	AGCAUUCAAACAUUCCCAAUUACC				8		
	osa-miR1854-3p	UCCAAUUUGGGGAUUUGCUGAU				8		

	osa-miR1854-5p	UGGUGAAAUUUGUAGAUUGGA				8		
	osa-miR1855	AGCACUGGAGUAGCCAAGAGA				11		
	osa-miR1856	UAUGCGUAAGACGGAUUCGUA	1			11		
	osa-miR1857-3p	UCAUGCUCCAAGAAAACCAGG	5	UUUUUGGAGCAUGAGGUUAUC		11		
	osa-miR1857-5p	UGGUUUUUUUGGAGCAUGAGG				11		
MIR1858	osa-miR1858a-b	GAGAGGAGGACGGAGUGGGGC			a	9		
					b	9		
	osa-miR1859	UUUCCUAUGACGUCCAUUCCAA	162			1	GGAAUGGAUGCGUAGAGAAAGA	22
	osa-miR1860-3p	AUCUGGAAGCUAGGUUUUCUCU	1	UUGUAGAUCUGGAAGCUAGGUUU		1		
	osa-miR1860-5p	AGAAAACCAGCUUCCAGAUCU				1		
MIR1861	osa-miR1861a	UGAUCUUGAGGCAGAAACUGAG	1	GAGGCAGAAACUGAGUAGUUGG[illegible]	a	1		
	osa-miR1861b,f,[illegible]	CGAUCUUGAGGCAGGAACUGAG	1	UCUUGAGGCAGGAACUGAGUA	b	2		
					f	5		
					i	6		
					l	9		
	osa-miR1861c	CGAUCUUGUAGCAAGAACUGAG			c	2		
	osa-miR1861d	UGGUCUUGAGGCAGGAACUGAG			d	4		
	osa-miR1861e,k,	CGGUCUUGUGGCAAGAACUGAG			e	4		
					k	8		
					m	9		
	osa-miR1861g	CAGUCUUGUGGCAAGAACUGAG			g	5		
	osa-miR1861h,j	CGGUCUUGAGGCAGGAACUGAG			h	6		
					j	8		
	osa-miR1861n	CGAUCUUGUGGCAGGAGCUGAG			n	12		
MIR1862	osa-miR1862a-c	ACGAGGUUGGUUUAUUUUGGGAC	18	UUGGUUUAUUUUGGGACGGAG	a	1		
MIR818					b	8		
					c	10		
MIR1862	osa-miR1862d	ACUAGGUUUGUUUAUUUUGGGAC	73			10		
MIR1862	osa-miR1862e	CUAGAUUUGUUUAUUUUGGGACG	87			11		
	osa-miR1863	AGCUCUGAUACCAUGUUAGAUUA[illegible]	21			2		

MIR1863	osa-miR1863b	AGCUCUGAUACCAUGUUAACUGUU				12
MIR1863	osa-miR1863c	UAGAAACUUGGCUGAUGCAUUAC	14			12
	osa-miR1864	UUGUAGUAACGUGAUGGUCAAUGU				2
	osa-miR1865-3p	CGAAGAAUCGCAGUCACUAGUUG	1	GAAGAAUCGCAGUCACUAGUUGU		3
	osa-miR1865-5p	UGCUAGUGAUGGUGAUUCUUCGAC				3
	osa-miR1866-3p	UGAAAUUCCUGUAAAAUUCUUG				3
	osa-miR1866-5p	GAGGGAUUUUGCGGGAAUUUCAC	1	UUGCGGGAAUUUCACGGGAAUUGA		3
	osa-miR1867	UUUUUUUUCUAGGACAGAGGGAG	16			3
	osa-miR1868	UCACGGAAAACGAGGGAGCAGCCA				4
MIR2118	osa-miR1869	UGAGAACAAUAGGCAUGGGAGGU	1	AUAGGCAUGGGAGGUAUUGGG		4
	osa-miR1870	UGCUGAAUUAGACCUAGUGGGCA	11	ATTAGACCTAGTGGGCATTTATAC		6
	osa-miR1870-3p	UUUAGGGCUAAUUCAGCAUGAACA				6
	osa-miR1871	AUGGCUCUGAUAUCAUGUUGGUU	1			6
	osa-miR1872	GAACUGUAAGUCUGUGACGGGUA	1(2)	GUUGAACUGUAAGUCUGUGACGGG		6
	osa-miR1873	UCAACAUGGUAUCAGAGCUGGAAG				7
	osa-miR1874-3p	UAUGGAUGGAGGUGUAACCCGAU	2			8
	osa-miR1874-5p	UAGGGCUACUACACCAUCCAUAAG				8
	osa-miR1875	ACAAUGGAGUGAAGUGCAACAGA	3	AAUGGAGUGAAGUGCAACAGAA		9
	osa-miR1876	AUAAGUGGGUUUGUGGGCUGGCC	58			10
	osa-miR1877	AGAUGACAUGUGAAUGAUGAGGGG				9
	osa-miR1878	ACUUAAUCUGGACACUAUAAAAG	2			9
	osa-miR1879	GUGUUUGGUUUAGGGAUGAGGUG	81			10
	osa-miR1880	UUCCAAGCGGGCCACUUAAGCAU	1	UUUUCCAAGCGGGCCACUUAAGCA		11
	osa-miR1881	AAUGUUAUUGUAGCGUGGUGGUGU				12
MIR1882	osa-miR1882a-h	AGAUUGCUUUCAAGGUCAUUUCUU			a	3
					b	-
					c	7
					d	8
					e	-
					f	11

					g	11		
					h	12		
MIR1883	osa-miR1883a-b	ACCUGUGACGGGCCGAGAAUGGA/	2	CCCGUCACAGGUAUCUAGUCAC(	a	11	AGGUGACUAGAUACCUGUGACGGG	2
					b	11		
MIR806	osa-miR1884a	UGUGACGCCGUUGACUUUUCAU	7		a	2	AAAAAGUCAACGGUGUCAUACA	1
MIR812	osa-miR1884b	AAUGUAUGACGCUGUUGACUUUUA			b	10		
	osa-miR2055	UUUCCUUGGGAAGGUGGUUUC	21			9		
	osa-miR2090	AACUCUGAUUCUAGAAUUUUUG				1		
	osa-miR2091-3p	CAUACAUUGCCUCCUAGGCUUG				12		
	osa-miR2091-5p	UCAACCGAGCCGAGGAGGAGG				12		
	osa-miR2092-3p	ACCAGCAUUCCAUUGGCAGAGG				12		
	osa-miR2092-5p	CAACUGAAGUCGGUGUUUACU				12		
	osa-miR2093-3p	ACAUCUUCCAAUUAAUGCAU				5		
	osa-miR2093-5p	GUGCAUUAAUUGGAAGAACA	7	ACUGGAUGUGUGCAUUAAUUGGAA		5		
	osa-miR2094-3p	CAGAGCUGUGGCAUCCACGUCG				2		
	osa-miR2094-5p	UGGCUGCUAGGCUCCUGGGUG	1	CGGGAGAAGUGUGGCUGCUAGGCU		2		
	osa-miR2095-3p	CUUCCAUUUAUGAUAAGUAU				10		
	osa-miR2095-5p	CUGAUAAUUUUACGAUGAAUAG				10		
	osa-miR2096-3p	CCUGAGGGGAAAUCGGCGGGA				1		
	osa-miR2096-5p	UGCCGAUUUCCCCCUCGGGCG	1	CGAUUUCCCCCUCGGGCGAGCC		1		
	osa-miR2097-3p	UUCUCUUCUUCGUGUCGCAUUU	1	CUCUUCUUCGUGUCGCAUUUCUUU		1		
	osa-miR2097-5p	AGAGAUGGGACGGGCAGGGAAG				1		
	osa-miR2098-3p	CGGUUUGUCAAGCGGAGUGC	1	UAACGGUUUGUCAAGCGGAGUGCA		10		
	osa-miR2098-5p	UCCCGUGGAGGCAGCCGAUG				10		
	osa-miR2099-3p	ACAAAGCUGUAGCGUUAUUC				1		
	osa-miR2099-5p	UGAAUAUGUUUGUACAAGCUUU				1		
	osa-miR2100-3p	AACCGCUGUUUAGGCGGAGUGG				11		
	osa-miR2100-5p	UUCUCUCAAGUUGCCAAACAAG	1	CCCUUCUCUCAAGUUGCCAAACAA		11		
	osa-miR2101-3p	AUUUAACUCAAGUGAGCAUUGU	16	CAAGUGAGCAUUGUGCGAGGCCAU		12		
	osa-miR2101-5p	ACAUGUUUACAAGUUAAAAUGU	5	AAGUUAAAAUGUGGAGCAGAGGGU		12		

	osa-miR2102-3p	CAUGGUGCCGGUUCCGGUGGCG				5
	osa-miR2102-5p	GGGCAAGCCGCCGCCGCCAC				5
	osa-miR2103	UUUCCCUCUCCGUGCGCGCUCG				12
	osa-miR2104	GCGGCGAGGGGAUGCGAGCGUG				11
	osa-miR2105	UUGUGAUGUGAAUGAUUCAU				12
	osa-miR2106	CCGAGGUUUUCUGGAUACAUU				2
	以上为探针所用数据库					
MIR2118	osa-miR2118a	UUCUCGAUGCCUCCCAUUCCUA			a	4
	osa-miR2118b,n	UUCCCGAUGCCUCCCAUUCCUA	2		b	4
					n	4
	osa-miR2118c,q	UUCCCGAUGCCUCCUAUUCCUA			c	4
					q	11
	osa-miR2118d	UUCCUGAUGCCUCCCAUGCCUA			d	4
	osa-miR2118e,r	UUCCCAAUGCCUCCCAUGCCUA			e	4
					r	11
	osa-miR2118f,j,r	UUCCUGAUGCCUCCCAUUCCUA			f	4
					j	4
					m	4
	osa-miR2118g	UUCCUAAUGCCUCCCAUUCCUA			g	4
	osa-miR2118h,k	UUCCUGAUGCCUCUCAUUCCUA			h	4
					k	4
	osa-miR2118i	UUCCUAGUGCCUCCCAUUCCUA			i	4
	osa-miR2118l	UUCCUAAUGCUUCCCAUUCCUA			l	4
	osa-miR2118o	CUCCUGAUGCCUCCCAAGCCUA			o	4
	osa-miR2118p	UUCCCGAUGCCUCCCAUGCCUA			p	11
	osa-miR2120	AAAGAUCUUUAGUCCCGGUUGUU(	5	AGAAAAGAUCUUUAGUCCCGGUUG		8
MIR2121	osa-miR2121a,b	AAAACGGAGCGGUCCAUUAGCGC(	7		a	1
					b	4
	osa-miR2122	UUUCAAAAAUAACCUUUUGUUC	16	AGACACCUCAGCGCCACGUCAACU		2
MIR806	osa-miR2123a,b,	UAAAAAGUCAACGGUGUCAAAC	3		a	4

					b	4		
					c	4		
MIR445	osa-miR2124a-i	ACUUUAAAUGUGUGUCCGUAUAU	1	UACUUUAAAUGUGUGUCCGUAU	a	2		
					b	5		
					c	7		
					d	7		
					e	8		
					f	10		
					g	11		
					h	11	AUCAAAUGUUUGGACACAUGCAUG	117
					i	11		
	osa-miR2125	UUUUUCUCUAUAGACUAUCUCCA	4	UUCUCUAUAGACUAUCUCCAGGUU		3		
MIR2275	osa-miR2275a	UUUGGUUUCCUCCAAUAUCUCA				8		
MIR2275	osa-miR2275b	UUUGGUUUCCUCCAAUAUCUCA				8		
	osa-miR2862	UCCAACAGCUUAGAUUCGUCC	5	AACAGCUUAGAUUCGUCCACGUCA		1		
MIR2863	osa-miR2863a	UUGUCCCAUUCUAGUUUAGCU				3		
MIR2863	osa-miR2863b	UUCGUUUAUUUGGACUAGAGU	1	UCGUUUAUUUGGACUAGAGUGGG		2		
	osa-miR2864.1	UUUUGCUGCCCUUGUUUUGCA				12		
	osa-miR2864.2	UUGUUUUGCAUUGUAUAGGUA				12		
	osa-miR2865	CUCAGCAGUCGACUGUACCGUG				9		
	osa-miR2866	UCUAGUUUGUGUUCAGCAUC				3	UGCUGAGCACAAACUAGAGAA	6
	osa-miR2867	UGUGCCAUCCCACACAUCCCGA				11		
	osa-miR2868	UUGGUUUUGUGUAGUAGAAA				11		
	osa-miR2869	UCCCGACAUUAAAUUCUGGGC	11	UUGGGAGGUGGUGAGUACUAAG		5		
	osa-miR2870	UAAUCAGUUUGGGGAGACAAA				1		
MIR2871	osa-miR2871a,b	UAUUUUAGUUUCUAUGGUCAC			a	5	GACCGUAGAAACUAGCAUAGAAA	1
					b	4		
	osa-miR2872	UGGGGUUCUACAAACCGAACU				11		
	osa-miR2873	AAGUUUGGACUUAAAUUUGGUAAC				11		
	osa-miR2874	AUGUGAACAGUGUCAAACAGUGUC				8		

	osa-miR2875	AUUUACAGUCAUAUACAGUUUAUA			8
	osa-miR2876	UUCCUAUAUGAACACUGUUGC			6
	osa-miR2877	UUGCAUCCUCUGCACUUUGGGCC	2	UUGGGCCUCGAGUGAUACAUUAAA	4
	osa-miR2878-3p	CAGGAUUUUAUACAUGUAAAGAAU			8
	osa-miR2878-5p	UACAUGUAUAAAAUUCUGAGGAU	1	AUGUAUAAAAUUCUGAGGAUGUUA	8
	osa-miR2879	GCCAGAUGUGUUAAAAUAAUGACC			2
	osa-miR2880	ACGGUAUCCCGUUCGGACAGGAU	1		3
	osa-miR2905	UACAUGUCAGUGACAAAGGCA			3
MIR2906	osa-miR2906a-b	AACGGGCCGCCGCACUGCUGG	1	AAGGAUAACGGGCCGCCGCACUGCUGG	3
					6
MIR2907	osa-miR2907a-d	GGCAGCCGAGCGAGGGCCUCGG	1	CACGCGGCAGCCGAGCGAGGGCCU	3
					11
					11
					12

*:The sequence and abundance of variants on the same locus was described in red characters

Additional file 3B The abundance of rice seed miRNAs from 3-12DAF in the library

miRNA family[a]	Sequence(5'-3')[b]	length	Abundance[c]	TPM[d]
osa-miR1866-3p	UGAAAUUCCUGUAAAAUUCUUG	22	0	0
osa-miR1846e	CAACGAGGAGGCCGGGACCA	20	0	0
osa-miR1428e-5p	UAAGAUAAUGCCAUGAAUUUG	21	0	0
osa-miR1846-5p	CAGUGAGGAGGCCGGGGCCGCU	22	0	0
osa-miR1874-5p	UAGGGCUACUACACCAUCCAUA	22	0	0
osa-miR2090	AACUCUGAUUCUAGAAUUUUUG	22	0	0
osa-miR2091-5p	UCAACCGAGCCGAGGAGGAGG	21	0	0
osa-miR2091-3p	CAUACAUUGCCUCCUAGGCUUG	22	0	0
osa-miR2092-5p	CAACUGAAGUCGGUGUUUACU	21	0	0
osa-miR2092-3p	ACCAGCAUUCCAUUGGCAGAGG	22	0	0
osa-miR2093-3p	ACACAUCUUCCAAUUAAUGCAU	22	0	0
osa-miR2093-5p	GUGCAUUAAUUGGAAGAACA	20	0	0
osa-miR2094-3p	CAGAGCUGUGGCAUCCACGUCG	22	0	0
osa-miR2094-5p	UGGCUGCUAGGCUCCUGGGUG	21	0	0
osa-miR2095-3p	CUUCCAUUUAUGAUAAGUAU	20	0	0
osa-miR2095-5p	CUGAUAAUUUUACGAUGAAUAG	22	0	0
osa-miR2096-3p	CCUGAGGGGAAAUCGGCGGGA	21	0	0
osa-miR2096-5p	UGCCGAUUUCCCCCUCGGGCG	21	0	0
osa-miR1850-5p.2	UUGUGUGUGAACUAAACGUGG	21	0	0
osa-miR1850-3p.2	CUGUUUAGUUCACAUCAAUCUU	22	0	0
osa-miR2097-3p	UUCUCUUCUUCGUGUCGCAUUU	22	0	0
osa-miR2097-5p	AGAGAUGGGACGGGCAGGGAAG	22	0	0
osa-miR2098-3p	CGGUUUGUCAAGCGGAGUGC	20	1	0
osa-miR2098-5p	UCCCGUGGAGGCAGCCGAUG	20	0	0
osa-miR2099-5p	UGAAUAUGUUUGUACAAGCUUU	22	0	0
osa-miR2099-3p	ACAAAGCUGUAGCGUUAUUC	20	0	0
osa-miR2100-5p	UUCUCUCAAGUUGCCAAACAAG	22	0	0
osa-miR2100-3p	AACCGCUGUUUAGGCGGAGUGG	22	0	0
osa-miR2101-3p	AUUUAACUCAAGUGAGCAUUGU	22	0	0
osa-miR2101-5p	ACAUGUUUACAAGUUAAAAUGU	22	0	0
osa-miR2102-3p	CAUGGUGCCGGUUCCGGUGGCG	22	0	0
osa-miR2102-5p	GGGCAAGCCGCCGCCGCCAC	20	0	0
osa-miR396f	UCUCCACAGGCUUUCUUGAACU	22	0	0
osa-miR2103	UUUCCCUCUCCGUGCGCGCUCG	22	0	0
osa-miR2104	GCGGCGAGGGGAUGCGAGCGUG	22	0	0
osa-miR2105	UUGUGAUGUGAAUGAUUCAU	20	0	0
osa-miR2106	CCGAGGUUUUCUGGAUACAUU	21	0	0
osa-miR1859	UUUCCUAUGACGUCCAUUCCAA	22	0	0
osa-miR827b	UUAGAUGACCAUCAGCAAACA	21	1	0

a,b:Name and sequence of miRNA data are from Xue et al., 2008.
c: abundance in the library ; d: the reads of miRNA are normalized in TPM = transcripts per million

Additional file 3C The abundance of rice seed miRNAs from 1-10DAF in the library

miRNA family[a]	Sequence(5'-3')[b]	length	Abundance[c]	TPM[d]
osa-miR1846a-c_5p	AGUGAGGAGGCCGGGGCCGCU	21	0	0
osa-miR1846d.2_3p	UAUCCGGCGCCGCAGGGAGG	20	0	0
osa-miR1847_5p	UGCAGUUUGCAGUUGUGGCAC	21	0	0
osa-miR1847_3p	UGGCCCACAUGUUAGUGCCACAAC	24	0	0
osa-miR1848	CCUCGCCGGCGCGCGCGUGCA	21	0	0
osa-miR1849	UAUCGUAUCCUAGGUUGGUUU	21	2	0
osa-miR1850	UGGAAAGUUGGGAGAUUGGGG	21	32	9
osa-miR1851d	CGUCUGGGAUGGCAUUUUGGC	21	0	0
osa-miR1852	AUAUGGAUUCAGAAUGCAGGU	21	0	0
osa-miR1853	UAAUUGGGGAUGUUCGGUUGC	21	0	0
osa-miR1854	UGGUGAAAUUUGUAGAUUGGA	21	1	0
osa-miR1855	AGCACUGGAGUAGCCAAGAGA	21	0	0
osa-miR1856	UAUGCGUAAGACGGAUUCGUA	21	1	0
osa-miR1857	UCAUGCUCCAAGAAAACCAGG	21	0	0
osa-miR1858a-b	GAGAGGAGGACGGAGUGGGGC	21	0	0
osa-miR1859	UUUCCUAUGACGUCCAUUCCAA	22	0	0
osa-miR1860	AUCUGGAAGCUAGGUUUUCUCU	22	1	0
osa-miR1861a	UGAUCUUGAGGCAGAAACUGAG	22	0	0
osa-miR1861b, f, i	CGAUCUUGAGGCAGGAACUGAG	22	0	0
osa-miR1861c	CGAUCUUGUAGCAAGAACUGAG	22	0	0
osa-miR1861d	UGGUCUUGAGGCAGGAACUGAG	22	0	0
osa-miR1861e, k, m	CGGUCUUGUGGCAAGAACUGAG	22	1	0
osa-miR1861g	CAGUCUUGUGGCAAGAACUGAG	22	0	0
osa-miR1861h,j	CGGUCUUGAGGCAGGAACUGAG	22	5	1
osa-miR1861n	CGAUCUUGUGGCAGGAGCUGAG	22	0	0
osa-miR1862a-c	ACGAGGUUGGUUUAUUUUGGGACG	24	0	0
osa-miR1862d	ACUAGGUUUGUUUAUUUUGGGACG	24	181	50
osa-miR1862e	CUAGAUUUGUUUAUUUUGGGACGG	24	122	34
osa-miR1863	AGCUCUGAUACCAUGUUAGAUUAG	24	2	0
osa-miR1864	UUGUAGUAACGUGAUGGUCAAUGU	24	0	0
osa-miR1865	UGCUAGUGAUGGUGAUUCUUCGAC	24	0	0
osa-miR1866	GAGGGAUUUUGCGGGAAUUUCACG	24	0	0
osa-miR1867	UUUUUUUUCUAGGACAGAGGGAGU	24	0	0
osa-miR1868	UCACGGAAAACGAGGGAGCAGCCA	24	0	0
osa-miR1869	UGAGAACAAUAGGCAUGGGAGGUA	24	0	0
osa-miR1870	UGCUGAAUUAGACCUAGUGGGCAU	24	0	0
osa-miR1871	AUGGCUCUGAUAUCAUGUUGGUUU	24	0	0
osa-miR1872	GAACUGUAAGUCUGUGACGGGUAA	24	0	0
osa-miR1873	UCAACAUGGUAUCAGAGCUGGAAG	24	0	0
osa-miR1874	UAUGGAUGGAGGUGUAACCCGAUG	24	0	0
osa-miR1875	ACAAUGGAGUGAAGUGCAACAGAA	24	0	0

osa-miR1876h	AUAAGUGGGUUUGUGGGCUGGCCC	24	0	0
osa-miR1877	AGAUGACAUGUGAAUGAUGAGGGG	24	0	0
osa-miR1878	ACUUAAUCUGGACACUAUAAAAGA	24	0	0
osa-miR1879	GUGUUUGGUUUAGGGAUGAGGUGG	24	0	0
osa-miR1880	UUCCAAGCGGGCCACUUAAGCAUU	24	0	0
osa-miR1881	AAUGUUAUUGUAGCGUGGUGGUGU	24	0	0
osa-miR1882a-h	AGAUUGCUUUCAAGGUCAUUUCUU	24	0	0
osa-miR1883a-b	ACCUGUGACGGGCCGAGAAUGGAA	24	0	0
osa-miR529b	AGAAGAGAGAGAGUACAGCUU	21	0	0
osa-miR531b	CUCGCCGGGGCUGCGUGCCG	20	0	0
osa-miR806i_as	UGUGACGCCGUUGACUUUUCAU	22	0	0
osa-miR806j_as	AAUGUAUGACGCUGUUGACUUUUA	24	0	0
osa-miR812f	ACGGAUGAUUAAAGUUGGACACGG	24	0	0
osa-miR812g-j	AAGACGGAUGAUUAAAGUUGGACA	24	0	0
osa-miR1423b_5p	CAACUACACGUUGGGCGCUCGA	22	0	0
osa-miR1428b, c, d	UAAGAUAAUGCCAUGAAUUCG	21	0	0
osa-miR1428e_3p	UAAGAUAAUGCCAUGAAUUUG	21	0	0
osa-miR1429.2	GUUGCACGGGUUUGUAUGUUGCAG	24	0	0

a,b:Name and sequence of miRNA data are from Zhu et al., 2008.

c: abundance in the library ; d: the reads of miRNA are normalized in TPM = transcripts per million
