# Supplementary material for: Identification of novel MiRNAs and MiRNA expression profiling during grain development in indica rice
Source: BMC Genomics. 2012 Jun 21;13:264. doi: 10.1186/1471-2164-13-264 (PMC3505464; doi:10.1186/1471-2164-13-264)
Supplement: Additional file 4 — Secondary structures of novel miRNAs. [file 1471-2164-13-264-S4.doc]

### Additional file 4. Secondary structures of novel miRNAs.

Predicted fold-back structures use the miRNA precursor sequences from rice. The EST sequences used for prediction of fold-back structures are indicated in parentheses. The expected novel miRNAs are shown in red letters, and the corresponding miRNA*s are shown in blue.

1. Can_miR -01 Initial dG = -67.60

---| U A AAG AAAAAC A AAAUGU U U

GUCCUAUUU AAGUGCA CUAU UUUUCGUGCC UUU AUCGUCUGUCUUAUUUG UUUUAUAAU AGUA U

CAGGGUAAA UUCACGU GGUA AAAGGCACGG AAA UAGCAGAUAGAAUAAAU AGAGUAUUG UUAU U

AGG^ - C CUA GUUA-- C AGU--- - U

GUCCUAUUUUAAGUGCAACUAUAAGUUUUCGUGCCAAAAACUUUAAUCGUCUGUCUUAUUUGAAAUGUUUUUAUAAUUAGUAUUUUUAUUGUUAUGAGAUGAUAAAUAAGAUAGACGAUCAAAAUUGGGCACGGAAAAUCAUGGCUGCACUUAAAUGGGACGGA

(((((((((.(((((((.((((...((((((((((...((.(((.(((((((((((((((((......(((((((((.((((....)))))))))))))...))))))))))))))))).))).)).))))))))))...)))).))))))))))))))))... (-68.70)

AAGUGCAACUAUAAGUUUUCGUGC +24 1 UUGGGCACGGAAAAUCAUGGC +21 1

UUGGGCACGGAAAAUCAUGGCUGC +24 6

ACGGAAAAUCAUGGCUGCACUUA +23 1

ACGGAAAAUCAUGGCUGCACUUAA +24 26

2. Can_miR -02 Initial dG = -55.30

--- C A CCA UG A-| U CCU

UUUGG UACUGGUA UCAAGUUAGGAA UGCAAGU UUUAUG GUUG GC \

GAACC GUGACCGU AGUUCAAUCUUU ACGUUCA AGAUAC CGAU CG C

UAAU U G AGA GU CG^ C UGU

GUUUGGCUACUGGUAAUCAAGUUAGGAACCAUGCAAGUUGUUUAUGAGUUGUGCCCUCUGUGCCUAGCGCCAUAGAUGACUUGCAAGAUUUCUAACUUGAGUGCCAGUGUCCAAGUAAUUUU

.(((((.((((((((.((((((((((((.(.(((((((..((((((.((((.((.......)).))))..))))))..))))))).).)))))))))))).)))))))).)))))....... (-56.50)

AAUCAAGUUAGGAACCAUGCAAGU +24 2

AAUCAAGUUAGGAACCAUGCAAGUU +25 3

AAUCAAGUUAGGAACCAUGCAAG +23 3

AUCAAGUUAGGAACCAUGCAAGUU +24 1

AUAGAUGACUUGCAAGAUUUCUAACUUG +28 1

UGACUUGCAAGAUUUCUAACUUGAGU +26 1

UUGCAAGAUUUCUAACUUGAGUG +23 1

3. Can_miR -03 Initial dG = -93.60

---------- - G A U A GC AAG .-CA| U

GUUUGAGGAUAUGUCAU UUUG UUUCC ACCC CCCGACUCUA AUGAACUAAG UC GAGUGA AAGUUCUU AUAUA A

CAAACUCCUAUACAGUA AAAC AAAGG UGGG GGGCUGAGAU UACUUGAUUC AG UUCACU UUCAAGAA UGUAU C

ACUUCUGGUA A - C C C GA --- \--^ T

GUUUGAGGAUAUGUCAUUUUGUUUCCGACCCACCCGACUCUAUAUGAACUAAGAUCGCGAGUGAAAGAAGUUCUUCAAUAUAUACUUAUGUCCUUGACCAACAUCGUAUCUCCCAUAAAUACGAAUCUCUACAUUAUCCUCGGUCCAUUCAAGAACUUUCACUUAGGACCUUAGUUCAUCUAGAGUCGGGCGGGUGGAAAACAAAAUGGUCUUCAAUGACAUAUCCUCAAAC

((((((((((((((((((((((((((.((((.((((((((((.((((((((((.((..(((((((...(((((((..(((((....)))))....((((....((((((.........)))))).....((...)).....)))).....))))))))))))))..)).)))))))))).)))))))))).)))))).))))))..........)))))))))))))))))) (-93.60)

ACUCUAUAUGAACUAAGAUCG +21 6

AUAUGAACUAAGAUCGCGAGUGAA +24 1

AUAUGAACUAAGAUCGCGAG +20 1

ACCUUAGUUCAUCUAGAGUCG +21 1

4. Can_miR -04 Initial dG = -69.10

AA A- A A UA CA .-AACUCCAA| CGC A

CGCU GA GA GAGAGC GGUGUAGCCAAGGA GACUUGCCGGCU GG UUCAGC \

GUGA CU CU CUCUUG CUACGUCGGUUCCU CUGAACGGCCGA CC GAGUCG A

C- CA C C CC A- \ --------^ UUA A

AACGCUAGAAGAAGAGAGCUAGGUGUAGCCAAGGACAGACUUGCCGGCUAACUCCAAGGCGCUUCAGCAAAGCUGAGAUUCCCCCAAUACCAGGAGCCGGCAAGUCAUCCUUGGCUGCAUCCCGUUCUCCUCCUCACAGUGC

..((((.((.((.((((((..((((((((((((((..((((((((((((........((...((((((...))))))...)).((.......)))))))))))))).))))))))))))))..)))))).)).))..)))). (-69.10)

AGAGAGCUAGGUGUAGCCAAG +21 1

CCGGCAAGUCAUCCUUGGCUG +21 32

UUGGCUGCAUCCCGUUCUCCUC +22 7

UGGCUGCAUCCCGUUCUCCUC +21 8

5. Can_miR -05 Initial dG = -56.50

AU----- UA - --| G G AC C CA C AAGAAA UG

GGGCGGUGC GGGUC GAAU UA CU CCG UCAUUCAC CA UGC AAGC CGCU A

UCUGCCGCG UCUAG CUUG AU GA GGC AGUAAGUG GU ACG UUCG GCGA G

GUUCGUU C- C CA^ G G GA A AG A AA---- UA

AUGGGCGGUGCUAGGGUCGAAUUAGCUGCCGACUCAUUCACCCACAUGCCAAGCAAGAAACGCUUGAGAUAGCGAAGCUUAGCAGAUGAGUGAAUGAAGCGGGAGGUAACGUUCCGAUCUCGCGCCGUCUUUGCUUG

..(((((((((..(((((((((((.((.(((..((((((((.((..(((.((((......((((......))))..)))).)))..)).))))))))..))).)).))..))).)))))).)))))))))....... (-56.50)

AGCUGCCGACUCAUUCACCC +20 11

AGCUGCCGACUCAUUCACCCA +21 30

AGUGAAUGAAGCGGGAGGU +19 1

AGUGAAUGAAGCGGGAGG +18 1

AGUGAAUGAAGCGGGAGGUA +20 1

AGUGAAUGAAGCGGGAGGUAA +21 29

6. Can_miR -06 Initial dG = -85.70

.-UUUUC A- U A UGG---- U-| UA

UUGG UCUCUC CUCCCUUGAAGGCU UCUCA AGGU UGAUG \

AACC AGAGAG GAGGGGGCUUCCGA AGAGU UCCG GUUAC C

\ ----- GA - A CAAAGAA UU^ CA

UUUUCUUGGAUCUCUCUCUCCCUUGAAGGCUAUCUCAUGGAGGUUUGAUGUACACCAUUGUUGCCUAAGAAACUGAGAAAGCCUUCGGGGGAGGAGAGAAGCCAA

...... ((((.(((((((((((((. (((((((((((((...((((((((((.....)))))))))))......))))))))))))))).)))))))))))))))))...

UCUCUCUCUCCCUUGAAGGCU +21 3

CUUCGGGGGAGGAGAGAAGC +20 1

7. Can_miR -07 Initial dG = -87.30

UAUAC| G GAA GU A A AACC A A

UA UACUUCCUCCGUUUCA UGUAAGACUUUCUAGCAUU C ACAUUCGUAUA AUGUUAAUG UA AUAUAU U

AU AUGAGGGAGGCAAAGU ACAUUCUGAAAGAUCGUAA G UGUAAGUAUAU UACAAUUAC AU UGUGUA A

GU---^ G AUA UG A C GUAA C U

UAUACUAGUACUUCCUCCGUUUCAGAAUGUAAGACUUUCUAGCAUUGUCAACAUUCGUAUAAAUGUUAAUGAACCUAAAUAUAUAUAUAUGUGUCUAAAUGCAUUAACAUCUAUAUGAAUGUAGGUAAUGCUAGAAAGUCUUACAAUAUGAAACGGAGGGAGUAGUAUG

.....((.((((((((((((((((...(((((((((((((((((((..(.(((((((((((.(((((((((....((.((((((....)))))).))....))))))))).))))))))))).)..)))))))))))))))))))...)))))))))))))))).)).. (-87.30)

UUUCUAGCAUUGUCAACAUUC +21 1

UUUCUAGCAUUGUCAACAUU +20 1

UUCUAGCAUUGUCAACAUUCG +21 2

UUCUAGCAUUGUCAACAUUCGUAU +24 1

UAUAUGAAUGUAGGUAAUGCUAGA +24 1

UAUGAAUGUAGGUAAUGCUAGAAA +24 1

AUGAAUGUAGGUAAUGCUAGAAAG +24 3

AAUGUAGGUAAUGCUAGAAAGUCU -24 1

AGGUAAUGCUAGAAAGUCUUA +21 1

UAAUGCUAGAAAGUCUUACAAUAU -24 1

AAUGCUAGAAAGUCUUACAAUAU -23 1

AAUGCUAGAAAGUCUUACAAU +21 3

8. Can_miR -08 Initial dG = -77.30

C ACAUU --- CC C U .-AUAAUU U U A| GA

CCGUCC AAAAUAA GCAU GAGUU CGUGUCCAACUUUGAUCGU CGUUUUAUUUGA UUUUUUU AGUAUU UUA UGUU UUA \

GGCAGG UUUUAUU CGUG UUCAA GCACAGGUUGAAACUAGUA GCAGAAUAAACU AAAAAAA UUAUGA AAU ACAA AAU U

A CGCGU GUA AA A U \ ------ U U -^ AU

CCGUCCCAAAAUAAACAUUGCAUGAGUUCCCGUGUCCAACUUUGAUCGUCCGUUUUAUUUGAUUUUUUUUAUAAUUAGUAUUUUUAUUGUUAUUAGAUUAUAAAACAUUAAUAGUAUUUUAUGCGUGACUCAUAUUUUUUAGUUUUCUCAAAAAAAAUUCAAAUAAGACGAAUGAUCAAAGUUGGACACGAAAACUUAUGGUGCUGCGCUUAUUUUAGGACGG

((((((.(((((((.(...(((((((((..(((((((((((((((((((.((((((((((((.(((((((.......((((...((((((((................))))))))....))))..((((.(......).))))......))))))).)))))))))))).)))))))))))))))))))..)))))...))))...).))))))).)))))) (-78.69)

CGUGUCCAACUUUGAUCGUCCGU +23 1

UGUCCAACUUUGAUCGUCCGUUUU -24 1

ACGAAUGAUCAAAGUUGGACACGA +24 1

GAAUGAUCAAAGUUGGACACGAA +23 8

GAAUGAUCAAAGUUGGACACGAAA +24 2

GAUCAAAGUUGGACACGAAAA +21 1

9. Can_miR -09 Initial dG = -81.40

AU C A C ---------| GACAUC A CUG GG C- UG AA-- U- C

UAG UAUGAA CUCAACAUGGUAUCAGA CUGGAAGU CUA AAGUU AUG UAGC ACGAU UGGAGA CA AU GC GAUAC \

GUC GUAUUU GAGUUGUACCAUAGUCU GAUCUUCA GAU UUCAA UAC AUCG UGUUA ACCUCU GU UA CG UUAUG A

\ CG U C A UCAAAAAGA^ ACAC-- - --- GA UU GU CAGC UU U

UAGAUUAUGAACCUCAACAUGGUAUCAGAACUGGAAGUCCUAAAGUUGACAUCAUGAUAGCCUGACGAUGGUGGAGACCAUGAUAAGCUGAUACCAUGUAUUUUGCCGACAUUGUGUUUCUCCAAGAUUGUGCUACAUCACAAACUUAGAAAAACUUAGAACUUCUAGCUCUGAUACCAUGUUGAGUUUUAUGGCCUGAUUAGU

(((..((((((.(((((((((((((((((.((((((((.((((((((......(((.((((...(((((..((((((.((..((..((.(((((...)))))..))....))..))..))))))..))))))))))))....)))).........)))).)))))))).))))))))))))))))).))))))..)))...... (-72.30)

ACCUCAACAUGGUAUCAGAACU +22 1

ACCUCAACAUGGUAUCAGAACUGG +24 8

UCAACAUGGUAUCAGAAC +18 1

UCAACAUGGUAUCAGAACUGG +21 1

UCAACAUGGUAUCAGAACUGGAAG +24 6

GCUCUGAUACCAUGUUGAGUUUU +23 1

10. Can_miR -10 Initial dG = -83.30

CGU - C A AAC A C CAAGU A AG

UUUUC GUUCGCCUUGAUCGCUAUUGACCUAG CAC CACGUGAUAU GU UCUACCC UCA ACA GU \

AAAAG CAGGCGGAACUAGCGAUAACUGGGUC GUG GUGUGCUAUA CA AGAUGGG AGU UGU CG A

--- A A C CUA A - AAC-- A UA

CGUUUUUCGUUCGCCUUGAUCGCUAUUGACCUAGCCACACACGUGAUAUAACGUAUCUACCCCUCACAAGUACAAGUAGAAUGCAUGUCAAUGAGGGUAGAAACAUCAUAUCGUGUGCGUGACUGGGUCAAUAGCGAUCAAGGCGGACAGAAAA

...(((((((((((((((((((((((((((((((.(((.((((((((((...((.(((((((.(((...(.(((.((.....)).))))..)))))))))).))...)))))))))).))).)))))))))))))))))))))))))).))))) (-82.30)

UUCGCCUUGAUCGCUAUUGAC +21 1

GCCUUGAUCGCUAUUGACC +19 1

GCCUUGAUCGCUAUUGACCU +20 2

UCAAUAGCGAUCAAGGCGGAC +21 3

GGUCAAUAGCGAUCAAGGCGGAC +23 1

CAAUAGCGAUCAAGGCGGAC +20 1

11. Can_miR-11 Initial dG = -83.20

C - AU-- C -| U A U CUG

UUGUUAAGAGUG UAAGUAUA AGUGUGUU UA GGGUAUU UGGU UUUUCCUGUCUCUUGUACAA AUAUAUUU \

AACAAUUCUCAU AUUCAUAU UCACACGA AU CCCAUAA ACCA GAAAGGGUAGAGAACAUGUU UAUAUAAA U

C G GAUC A C^ U G U UCG

CUUGUUAAGAGUGUAAGUAUAAUAGUGUGUUCUAGGGUAUUUUGGUAUUUUCCUGUCUCUUGUACAAUAUAUAUUUCUGUGCUAAAUAUAUUUUGUACAAGAGAUGGGAAAGGACCAUAAUACCCCUAAAGCACACUCUAGUAUACUUAGUACUCUUAACAAC

.((((((((((((((((((((..((((((((.(((((((((.((((.((((((((((((((((((((.((((((((.......)))))))).)))))))))))))))))))).)))).)))).))))).))))))))....)))))))).)))))))))))). (-83.20)

AGGGUAUUUUGGUAUUUUCCUGUC +24 1

AAUAUAUUUUGUACAAGAGAUGGG +24 10

UGGGAAAGGACCAUAAUACCCCUA +24 7
