# Supplementary material for: Identification of novel MiRNAs and MiRNA expression profiling during grain development in indica rice
Source: BMC Genomics. 2012 Jun 21;13:264. doi: 10.1186/1471-2164-13-264 (PMC3505464; doi:10.1186/1471-2164-13-264)
Supplement: Additional file 5 — Predicted targets of novel miRNAs. [file 1471-2164-13-264-S5.doc]

**Can_miR_01**

5’ GCACGAAAACUUAUAGUUGCACUU 3’ LOC_Os02g07960 (ATP binding protein)

||||| ||||| || | |||||||

3’ CGUGCCUUUGAGUACCGACGUGAA 5’ Can_miR_01-5p,3

**Can_miR_02**

none

**Can_miR_03**

5’ UGAUCUUAUUUCAUGUGGAGA 3’ LOC_Os01g50470 (ran GTPase binding protein)

||||||| ||||| | |||

3’ GCUAGAAUCAAGUAUAUCUCA 5’ Can_miR_03-5p,3.5

**Can_miR_04**

5’ GGUGGGAGCGGGAUGCGGCCGA 3’ LOC_Os11g18750 (hypothetical protein)

| | || |||||||| ||| |

3’ CUCCUCUUGCCCUACGUCGGUU 5’ Can_miR_04-5p,3.5

**Can_miR_05**

5’ UGCGUGAAAGAGUUGGUGGCU 3’ LOC_Os02g03240 (transposon protein)

|| ||||| |||| || |||

3’ ACCCACUUACUCAGCCGUCGA 5’ Can_miR_05-3p,3.5

5’ UGGUUGAGUGAUUCGGCGGUU 3’ LOC_Os04g52270 (coatomer subunit epsilon)

||| ||| ||| ||||| | |

3’ ACCCACUUACUCAGCCGUCGA 5’ Can_miR_05-3p,3.5

5’ UGGGUCAACGAGUCGGCGGCC 3’ LOC_Os09g16090 (cytokinin-O-glucosyltransferase 1)

||||| || |||||||| ||

3’ ACCCACUUACUCAGCCGUCGA 5’ Can_miR_05-3p,3.5

**Can_miR_06**

5’ GGCCUUUGAGGGAGAGAGAGG 3’ LOC_Os10g42400 (expressed protein)

||||| ||||||||||||

3’ UCGGAAGUUCCCUCUCUCUCU 5’ Can_miR_06-3p,2

5’ GGCCGUCGAGGGAGAGGGAGA 3’ LOC_Os06g42990 (AP2 domain containing protein)

||| || |||||||| ||||

3’ UCGGAAGUUCCCUCUCUCUCU 5’ Can_miR_06-3p,2.5

5’ GGCCUUGGAGGGAGGGAGAGG 3’ LOC_Os02g41810 (hypothetical protein)

||||| |||||| |||||

3’ UCGGAAGUUCCCUCUCUCUCU 5’ Can_miR_06-3p,3

5’ AGACUUCAAGGGAGAGGGGGC 3’ LOC_Os04g12060 (retrotransposon)

|| ||||||||||||| | |

3’ UCGGAAGUUCCCUCUCUCUCU 5’ Can_miR_06-3p,3

5’ AGGUUUCCGGGGAGAGAGAGA 3’ LOC_Os04g18244 (retrotransposon protein)

|| ||| ||||||||||||

3’ UCGGAAGUUCCCUCUCUCUCU 5’ Can_miR_06-3p,3

5’ AGUCUCCAAGGAAGAGAGGGA 3’ LOC_Os09g04260 (retrotransposon protein)

|| || ||||| |||||| ||

3’ UCGGAAGUUCCCUCUCUCUCU 5’ Can_miR_06-3p,3

5’ AGCUUUCGAGGGGGAAAGAGA 3’ LOC_Os10g30150 (ethylene-responsive protein)

||| ||| |||| || |||||

3’ UCGGAAGUUCCCUCUCUCUCU 5’ Can_miR_06-3p,3

5’ GUGUGUGAGGGAGAGAGAGA 3’ LOC_Os04g51190 (growth-regulating factor)

|: | ::|||||||||||||

3’ CGGAAGUUCCCUCUCUCUCU 5’ gn-06-3p 3.5

5’ GCCGGCGAGAGAGAGAGAGA 3’ LOC_Os08g06100 (quercetin 3-O-methyltransferase 1)

||| |:|| ||||||||||

3’ CGGAAGUUCCCUCUCUCUCU 5’ gn-06-3p 3.5

5’ GGCUUUUGAGGGGGAGAGAGU 3’ LOC_Os01g36910 (Transposable element protein)

|| || |||| |||||||

3’ UCGGAAGUUCCCUCUCUCUCU 5’ Can_miR_06-3p,3.5

**Can_miR_07**

5’ CUUUCUAGCAUUACCCACAUUCAU 3’ LOC_Os03g15033 (expressed protein)

||||||||||||||| ||||||||

3’ GAAAGAUCGUAAUGGAUGUAAGUA 5’ Can_miR_07-5p,1

5’ CUUUCUAGCAUUAUCUACAUUCGU 3’ LOC_Os07g09150 (expressed protein)

||||||||||||| |||||||| |

3’ GAAAGAUCGUAAUGGAUGUAAGUA 5’ Can_miR_07-5p,1

5’ CUUUCUAGCAUUAUCUACAUUCGU 3’ LOC_Os07g03110 (F-box domain containing protein)

|||||||||||||| ||||||||

3’ GAAAGAUCGUAAUGGAUGUAAGUA 5’ Can_miR_07-5p,1.5

5’ CUUUCUAGCAUUACCCACGUUCAU 3’ LOC_Os09g15639 (expressed protein)

||||||||||||||| || |||||

3’ GAAAGAUCGUAAUGGAUGUAAGUA 5’ Can_miR_07-5p,1.5

5’ CUUUCUAGCAUUGCCCACAUUCAU 3’ LOC_Os10g33700 (ATP binding protein)

|||||||||||| || ||||||||

3’ GAAAGAUCGUAAUGGAUGUAAGUA 5’ Can_miR_07-5p,1.5

5’ CUUUCUAGCAUUGCCCACAUUCAU 3’ LOC_Os12g40920 (light-inducible protein CPRF-2)

|||||||||||| || ||||||||

3’ GAAAGAUCGUAAUGGAUGUAAGUA 5’ Can_miR_07-5p,1.5

5’ CUUUCUAUCAUUGCCUACAUUUAU 3’ LOC_Os01g47680 (60S ribosomal protein)

||||||| |||| |||||||| ||

3’ GAAAGAUCGUAAUGGAUGUAAGUA 5’ Can_miR_07-5p,2

5’ CUUUCUAGUAUUGCCCACAUUCAU 3’ LOC_Os02g16490 (expressed protein)

|||||||| ||| || ||||||||

3’ GAAAGAUCGUAAUGGAUGUAAGUA 5’ Can_miR_07-5p,2

5’ CUUUCUAGCAUUGCCCACGUUCAU 3’ LOC_Os06g35590 (reticuline oxidase precursor)

|||||||||||| || || |||||

3’ GAAAGAUCGUAAUGGAUGUAAGUA 5’ Can_miR_07-5p,2

5’ CUUUCUAGUAUUGCCCACAUUCAU 3’ LOC_Os08g38620 (expressed protein)

|||||||| ||| || ||||||||

3’ GAAAGAUCGUAAUGGAUGUAAGUA 5’ Can_miR_07-5p,2

5’ CUUUUUAGCAUUGUCUAUAUUCAA 3’ LOC_Os08g39240 (cytoplasmic kinase)

|||| ||||||| ||| |||||

3’ GAAAGAUCGUAAUGGAUGUAAGUA 5’ Can_miR_07-5p,3

5’ UUUUCUAACAUUGCUUAUAUUCAU 3’ LOC_Os09g30050 (expressed protein)

|||||| |||| | || ||||||

3’ GAAAGAUCGUAAUGGAUGUAAGUA 5’ Can_miR_07-5p,3

5’- CTTTTTAATATTATCTATATTCAT -3’ LOC_Os02g51070 (soluble starch synthase 2)

||||:|| :||||:|||:||||||

3’- GAAAGATCGTAATGGATGTAAGTA -5’ Can_miR_07-5p,3

**Can_miR_08**

5’ UUCAUGUCCAGUUUUGAUAAUUC 3’ LOC_Os03g05040 (expressed protein)

||| |||||| |||||| ||||

3’ AAGCACAGGUUGAAACUAGUAAG 5’ Can_miR_08,3.5

### Additional file 5. Predicted targets of novel miRNAs

Predicted target(s) with a score of 3.5 or less are shown with alignments to the novel miRNAs.
