# Supplementary material for: Identification of novel MiRNAs and MiRNA expression profiling during grain development in indica rice
Source: BMC Genomics. 2012 Jun 21;13:264. doi: 10.1186/1471-2164-13-264 (PMC3505464; doi:10.1186/1471-2164-13-264)
Supplement: Additional file 6 — A Expression patterns of conserved miRNAs. [file 1471-2164-13-264-S6.doc]

| Additional file 6A. Expression patterns of conserved miRNAs | | | | | |
| --- | --- | --- | --- | --- | --- |
| Patterns | miRNA Name | p-value | Expression signal ± SD signal | | |
|  |  |  | G1^a^ | G2 | G3 |
| Up | osa-miR156a-j | 1.78E-01 | 1083±119 | 1408±157 | 1349±285 |
|  | osa-miR156k | 2.96E-01 | 875±567 | 1291±155 | 874±209 |
|  | osa-miR156l | 4.27E-02 | 581±228 | 1035±138 | 970±156 |
|  | osa-miR159a.1 | 2.91E-02 | 6803±1019 | 7361±275 | 9702±1438 |
|  | osa-miR159c | 2.81E-02 | 3150±1315 | 4971±573 | 6592±738 |
|  | osa-miR159d | 2.70E-02 | 3522±1272 | 5180±578 | 6973±893 |
|  | osa-miR159e | 2.89E-02 | 3237±1310 | 5008±619 | 6685±797 |
|  | osa-miR159f | 3.44E-02 | 5805±1424 | 7023±304 | 9193±1130 |
|  | osa-miR164a,b,f | 3.97E-02 | 407±90 | 385±52 | 928±443 |
|  | osa-miR164c | 5.62E-02 | 581±121 | 494±48 | 1053±435 |
|  | osa-miR164d | 3.92E-02 | 431±95 | 406±52 | 950±439 |
|  | osa-miR164e | 8.35E-02 | 803±156 | 659±69 | 1283±510 |
|  | osa-miR399d | 1.64E-01 | 154±39 | 171±9 | 222±56 |
|  | osa-miR399j | 1.20E-01 | 128±29 | 155±10 | 191±48 |
| Down | osa-miR160a-d | 8.01E-04 | 251±92 | 69±10 | 38±11 |
|  | osa-miR160e | 1.16E-03 | 275±114 | 68±8 | 48±9 |
|  | osa-miR160f | 1.27E-03 | 125±32 | 38±2 | 31±12 |
|  | osa-miR166a-d,f,n | 1.04E-02 | 1192±372 | 478±176 | 359±115 |
|  | osa-miR166e | 1.85E-02 | 494±125 | 242±68 | 157±71 |
|  | osa-miR166g-h | 1.86E-02 | 885±14 | 559±119 | 383±161 |
|  | osa-miR166i-j | 4.76E-02 | 208±114 | 138±25 | 49±40 |
|  | osa-miR166k-l | 5.42E-02 | 575±90 | 365±88 | 184±143 |
|  | osa-miR166m | 3.55E-02 | 844±56 | 589±141 | 403±173 |
|  | osa-miR167a-c | 3.11E-02 | 4338±919 | 3868±106 | 2900±286 |
|  | osa-miR167d-j | 4.21E-02 | 5270±1328 | 4295±178 | 3325±323 |
|  | osa-miR171a | 1.13E-03 | 118±19 | 58±13 | 42±6 |
|  | osa-miR171b-f | 4.64E-03 | 451±108 | 253±18 | 182±40 |
|  | osa-miR171h | 2.75E-01 | 38±34 | 33±4 | 17±8 |
|  | osa-miR396a-b | 2.07E-02 | 1395±727 | 1894±241 | 175±216 |
|  | osa-miR396c | 1.33E-01 | 366±592 | 500±169 | 44±35 |
|  | osa-miR396d-e | 5.64E-02 | 15297±3448 | 9823±1723 | 3931±3316 |
|  | osa-miR396f | 2.74E-02 | 21188±3929 | 12737±2543 | 6299±4348 |
| Others | osa-miR319a-b | 5.85E-01 | 409±29 | 364±131 | 322±126 |
|  | osa-miR162a | 4.34E-01 | 972±22 | 1478±255 | 1229±598 |
|  | osa-miR162b | 4.51E-01 | 508±11 | 666±150 | 500±209 |
|  | osa-miR168a | 2.88E-01 | 1643±531 | 1104±247 | 1201±271 |
| a: The first number is the mean value of signal and the second number is the square deviation value.  b: The first number is the mean value of signal and the second number is the square deviation value.  ^: The variant is located on the same precursor but is different from the known miRNA. | | | | | |

| Additional file 6B. Expression patterns of non-conserved miRNAs | | | | | |
| --- | --- | --- | --- | --- | --- |
| Patterns | miRNA Name | p-value | Expression signal ± SD signal | | |
|  |  |  | G1^a^ | G2 | G3 |
| Up | osa-miR535 | 3.75E-02 | 598±94 | 565±74 | 885±183 |
|  | osa-miR1850.1 | 7.40E-03 | 56±5 | 53±17 | 293±177 |
|  | osa-miR1862d | 3.17E-03 | 247±31 | 526±121 | 656±148 |
|  | osa-miR1862e | 3.69E-03 | 133±27 | 378±80 | 384±120 |
|  | osa-miR1866-5p | 4.20E-02 | 38±16 | 60±9 | 165±109 |
|  | osa-miR1874-3p | 3.57E-03 | 2473±698 | 4684±93 | 5507±424 |
|  | osa-miR1874-5p | 2.29E-05 | 349±27 | 1140±92 | 1266±220 |
|  |  |  |  |  |  |
| Down | osa-miR444a.1,d.1 | 5.71E-02 | 84±56 | 62±17 | 28±8 |
|  | osa-miR444a.2,d.2,e | 5.99E-02 | 481±33 | 363±61 | 205±139 |
|  | osa-miR444b.1,c.1 | 1.57E-02 | 241±22 | 168±13 | 77±27 |
|  | osa-miR444b.2,c.2 | 3.68E-04 | 770±60 | 443±146 | 63±29 |
|  | osa-miR444f | 6.79E-02 | 275±340 | 263±70 | 42±26 |
|  | osa-miR530-3p | 1.95E-02 | 279±121 | 124±15 | 84±24 |
|  | osa-miR530-5p | 4.18E-04 | 4522±635 | 3286±450 | 1514±306 |
|  | ^osa-miR1870 | 3.16E-02 | 104±25 | 93±2 | 57±25 |
|  | osa-miR2055 | 5.23E-03 | 138±13 | 140±11 | 97±11 |
|  |  |  |  |  |  |
| Others | osa-miR528 | 3.81E-02 | 1278±275 | 674±104 | 1975±943 |
|  | osa-miR529b | 1.94E-01 | 472±176 | 296±9 | 386±99 |
|  | ^osa-miR529 | 2.01E-02 | 930±174 | 406±65 | 644±249 |
|  | osa-miR1859 | 4.31E-02 | 29±16 | 158±29 | 82±57 |
| a: The first number is the mean value of signal and the second number is the square deviation value.  b: The first number is the mean value of signal and the second number is the square deviation value.  ^: The variant is located on the same precursor but is different from the known miRNA. | | | | | |

| Additional file 6C. Expression patterns of novel miRNAs and their miRNA* | | | |
| --- | --- | --- | --- |
| Reporter Name | Expression signal ± SD signal | | |
|  | G1^a^ | G2 | G3 |
| Can_miR01 | 48±9 ^a^ | 44±4 | 77±27 |
| Can_miR02 | 24±5 ^b^ | 41±4 | 33±13 |
| Can_miR03 | 24±8 | 63±20 | 28±12 |
| Can_miR04 | 19±3 | 16±2 | 21±13 |
| Can_miR05 | 88±17 | 82±15 | 232±135 |
| Can_miR05* | 39±7 | 49±7 | 73±11 |
| Can_miR06 | 66±7 | 69±15 | 110±39 |
| Can_miR06* | 58±8 | 37±3 | 33±4 |
| Can_miR10 | 140±39 | 123±18 | 154±41 |
| Can_miR11 | 44±6 | 37±2 | 18±5 |
| Can_miR11* | 20±3 | 20±2 | 32±14 |
| a: The a first number is the mean value of signal and the second number is the square deviation of signal.  b: The b numbers highlighted in yellow means miRNAs detected below or near the background average.  c: The probes with signal intensities ≤ 32 are considered as not expressing at this stage. | | | |
