# Supplementary material for: Identification of novel MiRNAs and MiRNA expression profiling during grain development in indica rice
Source: BMC Genomics. 2012 Jun 21;13:264. doi: 10.1186/1471-2164-13-264 (PMC3505464; doi:10.1186/1471-2164-13-264)
Supplement: Additional file 7 — Validation and expression of selected miRNA target genes. [file 1471-2164-13-264-S7.doc]

**Additional file 7. Validation and expression of selected miRNA target genes.**

1. **5’-RLM-RACE analysis of the cleavage of potential miRNA–target pairs in rice filling cereals**

5’-GUCUCGAGGGAAGUUAGGUUU-3’ miR159a.1

..||||||||||||o|||||.

3’-UGGAGCUCCCUUCACUCCAAG-5’ Os01g59660 (transcription factor GAMYB)

8/9

5’-UUCGAACUCUGUUGUUGACGU-3’ miR444b.2

|||||||||||||||||||||

3’-AAGCUUGAGACAACAACUGCA-5’ Os04g38780 (MADS-box transcription factor)

6/6

5’-CUAUAACCGUGCCGAGUUAGU-3’ miR171

|||||||||.|||||||||||

3’-GAUAUUGGCGCGGCUCAAUCA-5’ Os02g44360

8/10

5’-CCGUAUGUCCCUCGGUCCGU-3’ miR160

||||||||||||||||||||

3’-GGCAUACAGGGAGCCAGGCA-5’ Os04g43910 (Auxin response factor)

5/6

5’-ACCGUAUGUCCCUCGGUCCGU-3’ miR160

||o|||o||||||||||||||

3’-UGACAUUCAGGGAGCCAGGCA-5’ Os04g59430 (Auxin response factor)

4/6

5’-CCCUUACUUCGGACCAGGCU-3’ miR166

o||.|||||||||||||||.

3’-UGGGAUGAAGCCUGGUCCGG-5’ Os03g43930（HD-Zip transcription factor）

3/12 7/12

5’-UUUUUCAAACUGAAUUCUUU-3’ miR1435

||.|||||||||||||o|||

3’-AAGAAGUUUGACUUAAAAAA-5’ Os04g44354（UDP-glucuronosyl transferase）

7/12 2/12

5’-UCGGAAGUUCCCUCUCUCUCU-3’ Can_miR_06

|||.|||.||||.||o|||||

3’-AGCUUUCGAGGGGGAAAGAGA-5’ Os10g30150（ethylene-responsive protein）

3/10 1/10

**B. Quantitative RT-PCR analysis of target genes and their corresponding miRNAs (array results) at different developmental stages**
