# Supplementary material for: Identification of novel MiRNAs and MiRNA expression profiling during grain development in indica rice
Source: BMC Genomics. 2012 Jun 21;13:264. doi: 10.1186/1471-2164-13-264 (PMC3505464; doi:10.1186/1471-2164-13-264)
Supplement: Additional file 8 — miRNA variant patterns among grain developing stages. [file 1471-2164-13-264-S8.doc]

| Additional file 8. miRNA variant patterns among grain developing stages | | | |
| --- | --- | --- | --- |
| Name | 1-5 DAF to 6-10 DAF^a^ | 6-12 DAF to 13-17DAF^c^ | 13-17 DAF to 18-20 DAF^c^ |
| miR156 | up | up | down |
| miR159 | down (40/31)^b^ | up | up |
| miR164 | up | up | up |
| miR399 | down (10/9) ^b^ | up | up |
| miR535 | up | —^d^ | up |
| miR160 | up (29/35) ^b^ | down | down |
| miR166 | up | down | down |
| miR167 | up | down | down |
| miR171 | down (61/57) ^b^ | down | down |
| miR396 | up | down | down |
| miR444 | up | down | down |
| a: Data in 1-10DAF are from Zhu et al., 2008.  b: The abundance (1-5DAF/ 6-10DAF) listed is not high enough to define the pattern.  c: Data from the microarray chip results.  d: The expression levels between the two phase are nearly unchanged. | | | |
