# Supplementary material for: Identification of novel MiRNAs and MiRNA expression profiling during grain development in indica rice
Source: BMC Genomics. 2012 Jun 21;13:264. doi: 10.1186/1471-2164-13-264 (PMC3505464; doi:10.1186/1471-2164-13-264)
Supplement: Additional file 9 — 5′RLM-RACE primers used in this study. [file 1471-2164-13-264-S9.doc]

| **Additional file 9. 5’RLM-RACE primers used in this study.** | |
| --- | --- |
| Primer name | Primer sequence |
| Os01g59660 inner | TGAAGGGGTTGCTGCTGGAGACT |
| Os01g59660 outer | TGCAGGTAGGGATCAACTAACTCAG |
| Os04g38780 inner | ACGCAGCACGGAAGCAAGCAAGT |
| Os04g38780 outer | AAGCTCTTGAAGTTGTGCCTCTGTA |
| Os02g44360 inner | AAGGGTTTCGTCCAAATCAAG |
| Os02g44360 outer | TCAAGTACAAGAGTGACCTTCCTGG |
| Os04g43910 inner | AGGCGAGCGCGGCCGTGG |
| Os04g43910 outer | GCCGTGGACCTGTCGTCC |
| Os04g59430 inner | AAGAAGCTTCTGACACAAGCCTAC |
| Os04g59430 outer | TGACACAAGCCTACCTGACTCTCT |
| Os03g43930 inner | GCTCCATGTTATCCCTACGGGTA |
| Os03g43930 outer | GTAATGGTGGAACTATTGAGCTT |
| Os04g44354 inner | CAGTGTCACTCACTTAGGAGGTCAATA |
| Os04g44354 outer | ATAAATTTCCTGAAATTCTTGTGCG |
| Os10g30150 inner | CAAGAGCTGCTTTAGCTGAACTCAT |
| Os10g30150 outer | ATGCCACCACCATATCCAGGAAT |
