# Supplementary material for: Identification of novel MiRNAs and MiRNA expression profiling during grain development in indica rice
Source: BMC Genomics. 2012 Jun 21;13:264. doi: 10.1186/1471-2164-13-264 (PMC3505464; doi:10.1186/1471-2164-13-264)
Supplement: Additional file 10 — Quantitative RT-PCR primers. [file 1471-2164-13-264-S10.doc]

| **Additional file 10. Quantitative RT-PCR primers** | | |  |
| --- | --- | --- | --- |
| miRNA | target gene | Forward primer | Reverse primer |
| miR159 | Os01g59660 | CCCTTCACTCCAATATCCC | GGAGCCAAAGTCAACCTG |
| miR444 | Os04g38780 | ACTGGCTTATTTGGCGTTAT | TGGCAGGTCTTGAACATCTC |
| miR171 | Os02g44360 | TTGCTGTTAGCCTCCCTGTT | AGCCACGACAACCTTAGGAC |
|  | Os02g44370 | TGCGTGTTCCTCCTTGACTC | TGCCGTCCAAGCACCATA |
| miR160 | Os04g43910 | AAGAAGATCGACGACACCAAGC | GGACGACCATCGGAGTTGC |
|  | Os04g59430 | GGCGTCACAATAACATCACCT | TCATCATCTACACCCTCTGGAA |
| miR166 | Os03g01890 | CATTGAGCCCAAGCAGAT | ACAGCCTGAAGCCGTGAA |
| miR1435 | Os04g44354 | GAGAATAACTCTTACAAAGGAGCAG | GGGAACGGCACGAGAAGCA |
